# Contribution of tumor-derived extracellular vesicles in the establishment of the pre-metastatic niche: lessons learned from past experimentations and future directions

**DOI:** 10.1007/s10585-026-10396-z

**Published:** 2026-03-07

**Authors:** Laurence Blavier, Andjela Crnjac, Yves A. DeClerck

**Affiliations:** Department of Pediatrics, Cancer and Blood Diseases Institute, Children’s Hospital Los Angeles, University of Southern California, Los Angeles, CA 90027 USA

**Keywords:** Metastasis, Pre-metastatic niche, Extracellular vesicles, In vivo cancer models, Tumor-derived EV

## Abstract

Tumor-derived extracellular vesicles (TEVs) have been shown to actively contribute to the establishment of the pre-metastatic niche (PMN) through multiple mechanisms of action and the transfer of cargo material to host cells. Here, we report a review of 120 manuscripts published between January 2010 and April 2025 describing observations from *in vivo* experiments aimed at the examination of the contribution of TEVs to the PMN and the metastatic niche (MN). Whereas most of these publications reported observations made with models of exogenous administration of TEVs prepared *in vitro*, five publications used endogenous models of TEVs released by implanted tumors to track their fate and examine their role in the PMN. Breast and colon cancers and melanoma are the most common types studied, and lung, liver, bone, and lymph nodes are the most common sites of metastasis examined. Methods to isolate, characterize and label TEVs in exogenous models vary significantly with differential ultracentrifugation (DUC) being the most common method of isolation used, electron microscopy, nano tracking analysis (NTA) and western blotting being used for their characterization, and lipophilic dyes used for labeling. Immunodeficient and immunocompetent mice were used in the majority (94%) with some studies done in zebrafish. Single and multiple administrations of TEVs with doses ranging from 5 to 200 µg/mouse were reported. A review of the cells targeted by TEVs, the organs involved and the effects observed in the PMN and MN, led us to identify seven functional hallmarks: (1) Immune reprogramming, (2) Stromal cell activation, (3) Vascular permeability and angiogenesis, (4) Lymphatic remodeling and lymphangiogenesis, (5) Bone remodeling, (6) Extracellular matrix (ECM) remodeling and (7) Direct tumorigenicity. Altogether, these effects create an inflammatory and immunosuppressive microenvironment that favors tumor growth. Exogenous and endogenous models have both advantages and limitations and provide important complementary information. In both models, loss of function experiments aimed at determining whether TEVs are necessary for the formation of the PMN and MN represent an important challenge. Such experiments, however, will be critical to provide evidence that targeting TEVs could be a viable approach to inhibit or prevent metastasis in patients affected by cancer.

**Supplementary Information:**

The online version contains supplementary material available at 10.1007/s10585-026-10396-z.

## Introduction

Metastasis is a major cause of failure to treat cancer and despite significant advances in our understanding of its mechanisms it remains difficult to treat or to prevent. Considerable progress has been made in elucidating the mechanisms of metastasis, since 1889 when Stephen Paget proposed the “Seed and Soil” theory [1]. The validity of this theory was experimentally confirmed by Isaiah Fidler who -using the B16 murine melanoma model- demonstrated the importance of the organotropism of tumor cells in determining their metastatic behavior [2]. For long, the accepted scientific concept has been that tumor cells have the ability to colonize every organ but are only able to proliferate in organs providing a microenvironment that promotes angiogenesis, tumor cell proliferation and immune escape [3]. Experiments in many cancers demonstrated that metastatic tumor cells have the potential to reprogram the microenvironment at the site of metastasis to their advantage, a process described as the formation of the metastatic niche (MN) [4, 5]. However, in 2006 *R. Kaplan and colleagues* at Cornell University, using murine models of metastatic melanoma and lung carcinoma, challenged this concept by providing experimental evidence that bone marrow-derived cells (BMDC) colonize organs prone to metastasis prior to the homing of metastatic tumor cells. Thus, alterations in the microenvironment at metastatic sites can occur before and not after metastatic tumor cells arrive, a process described as the formation of the pre-metastatic niche (PMN) [6]. The establishment of the PMN is a complex, dynamic, and evolving process [7]. In its early stage, it is characterized by extracellular matrix (ECM) remodeling and inflammatory changes caused by tissue-resident immune and stromal cells. This stage is followed by an enhanced deposition of ECM proteins such as fibronectin and tenascin C, and an increase in vascular permeability that facilitates the recruitment of BMDC and later tumor cells. In its latest stage, the PMN is characterized by an infiltration of immunosuppressive myeloid cells (neutrophils and monocytes), tumor-associated macrophages (TAM) and endothelial progenitor cells that contribute to the formation of a myeloid-rich, lymphoid-poor immunosuppressive and pro-angiogenic microenvironment [8–11]. In a prospective study in patients with pancreatic cancer who had liver biopsy done at the time of surgical tumor resection, investigators provided indirect evidence for the formation of a PMN in patients with cancer. They reported that the liver of patients who later developed metastasis exhibited signs of augmented inflammation with an enrichment of neutrophil extracellular traps (NETs), Ki-67 upregulation and decreased liver creatine that significantly distinguished them from livers of patients who did not developed metastasis and the livers of non-cancer control patients [12].

The existence of a PMN raised the central question of the mechanisms by which tumor cells distantly communicate with cells in the future PMN to induce changes favorable for their implantation. The demonstration that the organotropism of metastatic tumor cells *in vivo* can be altered upon priming with the conditioned medium of tumor cells brought the attention to soluble factors including growth factors and cytokines [13] and extracellular vesicles (EVs) [14]. These latter, represent a heterogeneous group of small organelles with a lipid-bilayer membrane ranging in size from 50 nm to > 10 μm in diameter, and designated according to their biogenesis and size as exosomes (< 200 nm), ectosomes (200–400 nm), migrasomes (> 500 nm), apoptotic bodies (> 1 μm), or oncosomes (10 μm) [15, 16]. EVs are released by all living cells and serve as important vehicles in cell-to-cell communication. A unique characteristic of EVs is their cargo rich in a large variety of proteins, lipids, metabolites, and nucleic acids, reflecting the cells from which they originate. Methods to isolate, purify and characterize EVs are numerous, some more rigorous than others and they vary in the type of EVs they isolate [17].

There is abundant evidence that tumor-derived EVs (TEVs) represent one of the endocrine mechanisms adopted by tumor cells to trigger the formation of the PMN and the recruitment of immuno-suppressive BMDC to the PMN. However, such evidence has been hampered by technical limitations in the ability to track TEVs and document their capture in the PMN, and by the animal models used. In this article we performed a systematic review of fifteen years (2010–2025) of peer-reviewed scientific publications reporting the role of TEVs in metastasis and the establishment of the PMN in animal models. We identified 120 publications which were reviewed for the methods reported to purify, characterize, administer and track TEVs *in vivo*. Cancer types, metastatic sites, cells capturing TEVs and effects of TEV-capture on the PMN and/or MN were recorded and tabulated. Observations made on exogenous and endogenous models were summarized and the article ends by identifying the future challenges in targeting TEVs to inhibit or prevent metastasis in patients with cancer.

## Methods

### Search and selection of publications

Publications were selected using PubMed Advanced Search Builder with the following search queries: “(“extracellular vesicles“[Title/Abstract] OR “EV“[Title/Abstract] OR “exosomes“[Title/Abstract] OR “endogenous release“[Title/Abstract]) AND (“metastatic“[Title/Abstract] OR “metastasis“[Title/Abstract] OR “pre-metastatic“[Title/Abstract] OR “premetastatic“[Title/Abstract] OR “pre-metastasis“[Title/Abstract]) AND (“metastatic niche“[Title/Abstract] OR “pre-metastatic niche“[Title/Abstract] OR “premetastatic niche“[Title/Abstract] OR “MN“[Title/Abstract] or “PMN“[Title/Abstract]) NOT (Review[Publication Type])”. The range of years selected was January 1, 2010-April 15, 2025. The list obtained was further sorted by removing additional review papers, papers reporting a role for non-tumor derived EVs and papers reporting work solely done *in vitro*. Publications were organized in an excel spreadsheet format and chronologically organized by PMID (Supplemental Table 1). Three publications that did not contain in their title or abstract the key words used in our search, were however included in our list of 120 papers because of their relevance [14, 18, 19].

### Publication analysis

Each publication was examined for the following aspects: (1) Type of cancer and metastatic site(s) studied, (2) Method(s) to isolate TEVs in exogenous models, (3) Methods to characterize TEVs, (4) Methods to label and track TEVs *in vivo*, (5) Models of TEV administration/release, (6) Amount of TEVs administered, (7) Duration of the experiment, (8) Metastatic model (immunocompetent vs. immunodeficient, experimental vs. orthotopic model), (9) Cells capturing TEVs, and (10) Effect of TEVs on the PMN or MN. The data were recorded and sorted using Microsoft Excel data collection and graphed using GraphPad Prism and BioRender.

## Results

### Exogenous models

**Fig. 1:**
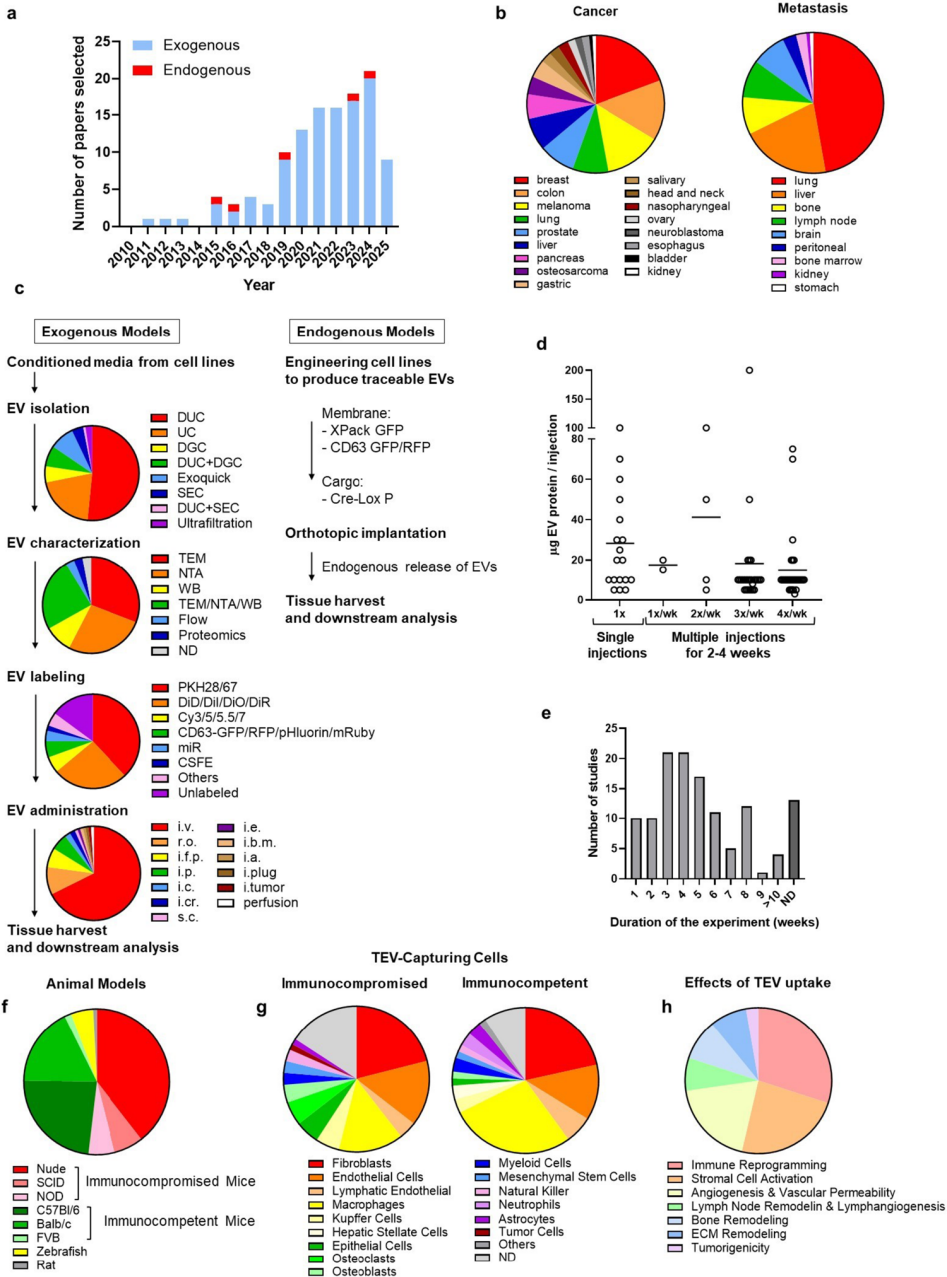
**a** Research papers published over the last fifteen years describing a role of TEVs in the pre-metastatic niche. **b** Pie diagrams representing the proportion of papers published per cancer models studied (left) and the proportion of papers addressing the listed metastatic sites (right). **c** Comparison between methods used for exogenous administration of TEVs versus the methods used in models of endogenous release of TEVs. Diagrams represent the proportion of papers using the listed techniques of TEV isolation, characterization, labeling, and administration. **d** Comparison of doses of TEV protein used in single and multiple administrations. **e** Duration of the experiment to analyze PMN and MN. **f** Proportions of studies done using different animal models, including immunocompromised vs. immunocompetent mice general backgrounds. **g** Proportions of studies identifying the listed TEV-capturing cells. **h** Proportions of studies identifying the listed hallmark effects of TEV-capture on the PMN and MN. DUC, differential ultracentrifugation; UC, ultracentrifugation; DGC, density gradient centrifugation; SEC, size exclusion chromatography; TEM, transmission electron microscopy; NTA, nano tracking analysis; WB, western blot; i.v., intravenous; r.o., retroorbital; i.f.p., intra food pad; i.p., intraperitoneal; i.c., intracardiac; i.cr., intracranial; s.c., subcutaneous; i.e., intra ear; i.b.m., intra bone marrow; i.a., intra anal

Our research identified a total of 120 peer reviewed original publications reporting a contribution of TEVs to metastasis between 2010 and 2025 with a significant increase after 2018. Among those, 115 used an exogenous model (Fig. 1a and Supplemental Table 2). These publications cover a large variety of cancer types with a majority being breast and colon cancers, and melanoma. Among the metastatic organs studied, lung, liver, bone, and lymph nodes were the most commonly examined with few reports on bone marrow, brain and kidney metastasis (Fig. 1b and Supplemental Table 3). The methods used in these models and the effects observed in the PMN and MN are summarized below. In exogenous models, TEVs were isolated, characterized, labeled, and administered using a large variety of methods (Fig. 1c). Differential ultracentrifugation (DUC) was the most common method used (56% of the reports) followed by ultracentrifugation (UC) (17% of the reports). OptiPrep™ density gradient centrifugation (ODGC) or sucrose density gradient centrifugation were used in 11% and size exclusion chromatography (SEC) in 4%. In 8% of the reports, a combination of two techniques (UC or DUC and SEC in 0.9% or UC/DUC and density gradient in 7%) was reported. Precipitation methods like ExoQuick^®^, were used in 9%. As a result, some of these publications refer to exosome whereas others, and particularly the most recent use the term EV. Among the 115 publications using exogenous models, 95% provided a detailed characterization of the material collected including transmission electron microscopy (TEM), or nano tracking analysis (NTA) to determine the size of the particles, and Western Blot (WB) to demonstrate the presence of proteins typically associated with EVs such as tetraspanins. A combination of these three methods was used in 35% of the papers, mostly in the most recent years in compliance with updated guidelines from the International Society of Extracellular Vesicles [17]. In 81% of the cases, TEV labeling was used as a second step in the 

preparation of TEVs to allow their tracking *in vivo*. Lipophilic fluorescent dyes like Paul Karl Horan (PKH, 36 %) dyes, carbocyanine dyes (DiD, Dil, DiO, or DiR, 24 %), or near infra-red fluorescent dyes Cy5.5 or Cy7 (5 %) were commonly used. In 62 % of those cases, TEV labeling was followed by density gradient centrifugation to remove excess dye. The routes of exogenous administration of TEVs differ among the publications reviewed. Intravascular systemic administrations were common routes (77.1 % intravenous and 1.9 % intraarterial), whereas local administration was used in 21 % of the studies. Doses and frequency of TEV administration widely ranged from 5-100 μg/mouse of TEV proteins in a single administration to 5-200μg/mouse of TEV proteins given 1-4 times per week over 2-4 weeks (Fig. 1d). Single injections (30%) were typically used to assess biodistribution of the TEVs, whereas multiple administrations (70%) were used to educate the PMN prior to the administration of cancer cells. In the five publications that used endogenous release of TEVs, tumor cells were engineered with a chimeric luminescent (luciferase) or fluorescent protein (GFP or RFP-tetraspanin CD63 or GFP-link kinase (XPack)) and the tracking of labeled TEVs was performed in animals implanted orthotopically. In all papers examined, the duration of the experiment required to analyze the PMN varied greatly, from one up to more than 10 weeks (Fig. 1e). A murine model was used in 94.1% of the studies with 55.4 % being done in immunodeficient and 44.6% in immunocompetent mice. Six reports used a Zebrafish model for its convenient transparency ideal for the tracking of TEVs and tumor cells. One publication reported a study done on rats (Fig. 1f). The different cell types capturing TEVs were similar in immunocompromised and immunocompetent animals, with the majority being identified as macrophages, fibroblasts, and endothelial cells (EC) (Fig. 1g). 

### The organotropism of TEVs is directed by adhesion molecules and TEV-capturing cells are organ specific

In a seminal paper, *Hoshino et al.* used PKH and near infra-red-labeled TEVs from MDA-MB-231 human breast cancer cells and subclones with organotropism for bone, lung, and brain, as well as TEVs from colorectal and pancreatic cancer cells metastatic to the liver, and demonstrated that TEVs have specific organotropism for these organs that is controlled by integrins with TEVs containing integrins α_6_β_4_ and α_6_β_1_ seeding in the lung and being associated with lung metastasis, while TEVs containing integrin α_v_β_5_ being retained in the liver and associated with liver metastasis. These TEVs are captured by resident cells mainly Kupffer cells (KC) in the liver, EC in the brain and fibroblasts and epithelial cells in the lung [20]. Another study has shown that TEVs from urological cancer cells injected intravenously are taken up within 12 h by the lung, liver, brain and spleen but not by other organs and that the level of incorporation in these organs depends on the type of cancer, with the lung being the dominant organ in prostate and bladder cancers and the brain in kidney cancers [21]. In breast cancer, the expression of the melanoma cell adhesion molecule (MCAM/CD146/MUC18) in TEVs allows them to be targeted to the lung [22].

### Seven hallmarks of TEVs

**Fig. 2:**
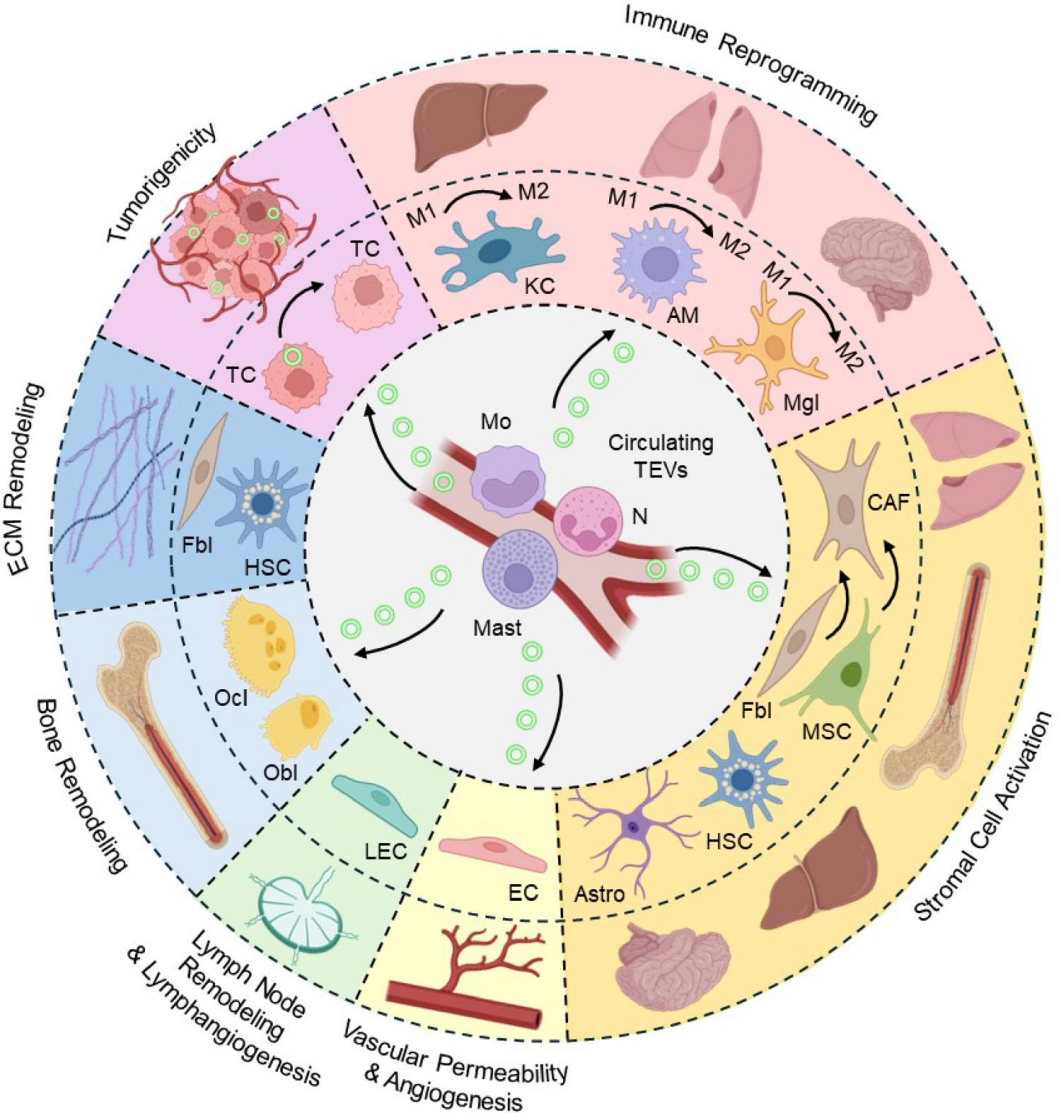
The seven hallmark effects of TEV-uptake on PMN and MN. KC, kupffer cell; AM, alveolar macrophage; Mgl, microglial cell; CAF, cancer associated fibroblast; Fbl, fibroblast; MSC, mesenchymal stromal cell; HSC, hepatic stellate cell; Astro, astrocyte; EC, endothelial cell; LEC, lymphatic endothelial cell; Obl, osteoblast; Ocl, osteoclast; TC, tumor cell; M, monocyte; N, neutrophil; Mast, mast cell

The uptake of TEVs has a broad spectrum of effects on the PMN and MN that is a function of the cell type and the organ being targeted (Fig. 1h). These effects are summarized here in seven functional categories, designated hallmarks (Fig. 2): (1) Immune reprogramming, (2) Stromal cell activation, (3) Vascular permeability and angiogenesis, (4) Lymphatic remodeling and lymphangiogenesis, (5) Bone remodeling, (6) ECM remodeling and (7) Direct tumorigenicity.

#### TEVs reprogram immune cells

TEVs are captured by immune innate cells like macrophages, monocytes, neutrophils, and mast cells which results in the reprogramming of their immune function often but not always toward an immune suppressive role. TEVs are preferentially taken by resident macrophages but also bone marrow-derived and circulating monocytes. Macrophages from the liver and KC capture TEVs from colorectal cancer (CRC) cells and gastric cancer cells which polarize them into M2 macrophages promoting metastasis [23–26]. TEVs containing angiopoietin like protein-1 taken by KC in the liver decrease the secretion of matrix metalloproteinase (MMP)-9 and improve vascular integrity [27]. In the lung, TEVs promote an immunosuppressive activity in macrophages by upregulating their expression of program death ligand (PDL)-1 resulting in T cells suppression and exhaustion [28, 29]. Such M2 polarization occurs through activation of several signaling pathways like ERK1/2, PI3K/AKT/mTOR, STAT3, YAP and TLR-NFkB [25, 30–33]. As a result, polarized macrophages secrete cytokines and chemokines like CCL2 [34], CXCL1 [35], IL-6 [36] and IL-8 [37, 38] attracting innate immune myeloid cells to the PMN such as myeloid derived suppressive cells (MDSC) and neutrophils [39, 40]. TEV-capturing macrophages (and lymphatic EC) can also act as tumor antigen-presenting cells inducing T cell apoptosis [41]. In the brain, microglial cells capture TEVs which promote their M2 polarization by regulating a miR-142p /TGFβ pathway that facilitates brain metastasis [42]. Peritoneal macrophages capturing TEVs play a role in the dissemination of ovarian and gastric cancers as they polarize toward M2 and secrete CCL2, CXCL5 and CCL5 [43, 44]. Bone marrow-derived monocytes capture TEVs in the bone marrow and the blood circulation and mature into M2 macrophages [45, 46]. However, some, like Ly6C^low^ patrolling monocytes, can have an inhibitory effect on metastasis as they capture TEVs from low metastatic cells and inhibit the growth of highly metastatic cells [47, 48]. A particular aspect of macrophages is their ability to transfer TEVs they have captured to other cells. They transfer TEVs to EC promoting angiogenesis [49] and to fibroblasts [50] stimulating tumor cell invasion. In immunodeficient mice, TEVs from osteosarcoma cells promote the recruitment of CD11b myeloid cells to the lung however without forming a functional PMN [51]. Mast cells, which play a promoting role in melanoma metastasis, capture the DNA-binding protein high mobility group AT-hook 1 (HMGA1) secreted by melanoma cells which contribute to their pro-metastatic activity [52]. Like macrophages, neutrophils capture circulating TEVs in the lung which activates TLR/NFkB signaling, the production of IL-1β [53] and their N2 polarization [54].

#### TEVs activate stromal cells

Fibroblasts capture TEVs which causes their activation into cancer-associated fibroblasts (CAF). In the liver, TEVs from CRC and hepatocellular carcinoma (HCC) are taken by fibroblasts in metastatic organs and hepatic stellate cells (HSC) in the liver which results in their activation into CAFs through a variety of regulatory mechanisms including miR-controlled protein phosphorylation [55], alteration in lipid metabolism [56] and the production of inflammatory extracellular proteins like Netrin1 [57] and cytokines like IL-6 and IL-8 [58–60]. Studies in lung fibroblasts also demonstrated that TEVs from a large variety of cancers convert fibroblasts into CAFs by activating NFkB and stimulating the production of inflammatory cytokines including IL-6, IL-8 and IL-11 [61–68] and inducing vascular leakiness [69]. TEVs from ovarian carcinoma activate adipose-derived mesenchymal stromal cells (MSC) into CAFs favoring omental metastasis [70, 71]. Fibroblasts capturing TEVs interact with T cells by secreting CCL1 which promotes their differentiation into immunosuppressive Treg [72]. In the brain, astrocytes that can differentiate into fibroblasts, capture melanoma TEVs which activate a proinflammatory signaling and the production of IL-6, IL-8, MCP-1 and CXCL1 promoting brain metastasis [68, 73]. TEVs also stimulate the secretion of IL-1β by astrocytes which induces T cell differentiation into pro-metastatic Th17 cells [74]. The capture of TEVs by epithelial cells is only reported in one publication where it induces the secretion of CCL2 via AKT/NFkB signaling promoting the recruitment of MDSC to the PMN [75].

#### TEVs stimulate angiogenesis and promote vascular permeability

Microvascular EC are at the frontline in capturing circulating TEVs. Numerous studies in murine and zebrafish models demonstrated that the uptake of TEVs by EC in the microvasculature increases vascular permeability and EC proliferation, creating an abnormal vasculature that facilitates the extravasation of immune cells and tumor cells. TEVs disrupt the formation of tight and adherens junctions between EC by inhibiting the production of cell-cell adhesion molecules such as zona occludens 1, claudin and occludin [76–83] and stimulate endothelial to mesenchymal transition [84]. VE-cadherin is decreased in EC upon capture of TEVs as it is either degraded by a TEV-induced production of the sheddase ADAM-17 [85] or is less expressed [86]. Upon TEV capture, EC initiate ECM remodeling through the production of VEGF, and MMP-2 and MMP-9 [87] and their increased expression of the receptor for urokinase plasminogen activator [88]. TEVs induce in EC the production and secretion of VEGF and chemokines like CX3CL1 (Fractalkine), or CCL2 recruiting immune cells to the PMN [89–91]. In the brain, the capture of TEVs by brain microvascular EC causes a disruption of the blood brain barrier facilitating its crossing by immune and tumor cells [79, 92]. TEV-capturing brain EC affect polarization of microglial macrophages into M2 via Dkk-1 [93]. The mechanisms involved in these profound alterations of the microvasculature are partially known and include a rerouting of the incorporated TEVs from lysosome degradation to endosome processing promoting the expression of angiogenic factors [94] and the activation of signaling pathways such as ERK1/2, AKT and NFkB [79, 89, 95].

#### TEVs remodel lymph nodes and stimulate lymphangiogenesis

TEVs have a specific organotropism for lymphatic EC. Homing of melanoma exosomes to sentinel lymph nodes results in ECM deposition, lymphatic EC proliferation and melanoma cell recruitment [19]. Through their content in adhesion molecules like α6 integrin [96], CD97 (a G protein coupled receptor) [97] and the nerve growth factor receptor NGFR/p75NTR [98] TEVs promote lymphangiogenesis and lymph node metastasis. The capture of TEVs by lymphatic EC increases their expression of adhesion molecules like ICAM, promoting the homing of circulating tumor cells (CTC) to lymph nodes [98]. Lymphatic EC can act as tumor antigen-presenting cells inducing T cell apoptosis [41]. TEVs are also captured by fibroblastic reticular cells in lymph nodes enhancing PDL-1 expression and T cell exhaustion [99]. TEVs taken by lymphatic EC promote the formation of NETs in the lymph node PMN [100]. The capture of TEVs by macrophages in lymph nodes enhances their secretion of VEGFC stimulating lymphangiogenesis [101].

#### TEVs remodel the bone

In the bone, TEVs promote an osteolytic PMN and MN through a variety of mechanisms. TEVs and large oncosomes captured by bone marrow macrophages and osteoclasts promote their activation and differentiation *in vitro* and their osteolytic activity *in vivo.* They activate in these cells signaling pathways such as NFkB, MAPK, N-WASP and MAFB resulting in an increase in osteolysis [102–107]. TEVs are also captured by osteoblasts stimulating secretory autophagic pathway, increasing inflammation and causing bone remodeling promoting breast and prostate cancer metastasis [108–111]. TEVs are taken by bone marrow MSCs enhancing glycolysis (reverse Warburg effect) and promoting the growth of metastatic cancer cells in the bone marrow [112].

#### TEVs remodel the ECM

The capture of TEVs by fibroblasts and stromal cells in the PMN and MN remodels the ECM increasing the deposition of ECM proteins like fibronectin, collagen, and tenascin C [113–116]. In an extensive *in vivo* study of liver metastasis in pancreatic ductal adenocarcinoma (PDAC), investigators demonstrated that TEVs are captured by KC and induce the expression and release of TGFβ which in turn increases the production of fibronectin by HSC in a MIF-dependent mechanism [117]. Other studies have shown that TEVs, in a hypoxic microenvironment, increase the cross linking of ECM proteins via their content in cross linking factors such as lysyl oxidase-like 2 and transglutaminase-2 [115, 118]. ECM remodeling involves not only the synthesis, deposition, and cross-linking of ECM proteins but also their degradation by MMPs secreted by TEV-capturing cells [119, 120].

#### TEVs enhance the metastatic behavior of tumor cells

TEVs are also captured by tumor cells in a paracrine mechanism. TEVs from high metastatic osteosarcoma cells are captured by low metastatic cells and induce a migratory and invasive phenotype [121] and ADAM-17 in the TEVs of CRC cells cleaves E-cadherin in surrounding tumor cells promoting epithelial to mesenchymal transition and migration [122].

### Endogenous models

More recently, innovative labeling strategies and reporter systems that can track TEV release and their capture in mice and zebrafish have been reported [123–126]. Some of these techniques have been combined with endogenous models of TEV release to study their role in the development of the PMN and MN.

In mice bearing genetically modified B16F10 melanoma tumors which produce TEVs carrying membrane-bound Gaussia luciferase, *Pucci et al.*, combined imaging and genetic analysis to track endogenously released TEVs at organismal, cellular, and molecular scales. They show that TEVs efficiently disseminate via lymphatics and preferentially bind to CD109^+^ macrophages in the subcapsular sinus of lymph nodes where they are blocked, indicating that these macrophages have an anti-tumorigenic activity. This blockage however is disrupted upon tumor progression or treatment with chemotherapy (paclitaxel or carboplatin) or immunotherapy (anti-CSFR1). They further demonstrate that in the absence of CD109^+^ macrophages, TEVs interact with B cells fostering a tumor-promoting humoral immunity [18].

Using an elegant Cre-Lox P system in which the capture of TEVs by cells was imaged *in vivo* by their change in fluorescence (from red to green), *Zomer et al.* provided evidence for a tumor cell-to-tumor cell TEV transfer process during which TEVs released by malignant cells are taken by less malignant tumor cells located within the same primary tumor and within distant tumors. They show that these TEVs carry mRNAs involved in migration and metastasis [124]. Using PC3 cells stably transduced with a lentivirus containing a fusion of CD63-GFP (PC3-ExGFP) injected into the prostate of male immunodeficient mice, *Dai et al.* [127] demonstrated the presence of green fluorescence in bone marrow stromal cells three weeks after orthotopic implantation of PC3 cells in the absence of tumor cells, providing the first evidence in an endogenous model in support for the central role of TEVs in triggering the PMN. Using two metastatic models of melanoma and neuroblastoma in immunodeficient mice implanted orthotopically with tumor cells engineered to release GFP-labeled TEVs, our laboratory demonstrated for the first time the capture of TEVs endogenously released in an organotropic manner with a capture in the lung in melanoma and in the liver in neuroblastoma by resident macrophages. As in the report of *Dai et al.*, we observed the presence of TEVs captured by macrophages prior to colonization by tumor cells. The capture of these TEVs by macrophages induced dynamic changes in inflammatory gene expression which evolved to a pro-tumorigenic reaction as the PMN progresses toward the MN [128]. Using an endogenous model of melanoma in immunocompetent mice, *Ortiz et al.* showed that melanoma TEVs downregulate type I interferon (IFN) receptor and expression of IFN-inducible cholesterol 25-hydroxylase (CH25H). CH25H produces 25-hydroxycholesterol, which inhibits TEV uptake. Mice incapable of downregulating the IFN receptor were resistant to TEV uptake. Treatment of mice with reserpine which decreased the release of TEVs in the plasma of tumor-bearing mice inhibited the formation of lung metastasis. The capture of TEVs in the PMN however was not examined as the TEVs were not labeled [129].

**Table 1:**
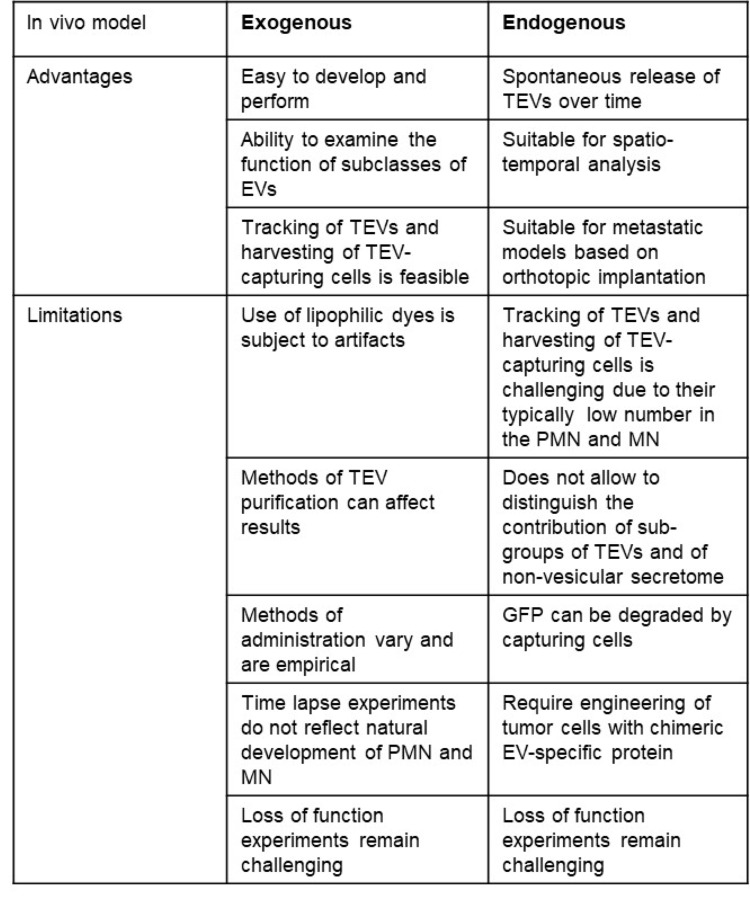
Comparison and complementarity between exogenous and endogenous models of TEVs

## Conclusion

This review of the literature of the last 15 years demonstrates that exogenous and endogenous models have been complementary in providing experimental evidence that TEVs have an important function in the formation of the PMN and the development of metastasis (Table 1). 

Exogenous models have the advantage of their simplicity and the standardization of their methodologies. The high number of TEV-capturing cells that can be harvested and analyzed is another advantage. Exogenous models also have the potential to identify specific subpopulations of TEVs that contribute to metastasis. These models, however, have limitations, particularly due to technical issues related to their *in vitro* isolation, the methods used for their purification and for their labeling. In particular, the use of lipophilic dyes can result in the formation of aggregates, or micelles, which could mimic EVs and therefore be counted as false-positive signals leading to an overestimation of EV uptake [130–132]. In addition, the doses and schedules used – as demonstrated by our review - widely vary and remain empirical. Endogenous models have the advantage to mimic the natural development of the PMN and its evolution toward the MN as they rely on tumors orthotopically implanted in mice. They are suitable for spatial-temporal analysis of TEVs capture and its effect on the PMN and evolution toward the MN. This advantage has, however, its own limitations due to the implantation of a large number of tumor cells or a tumor fragment. In this regard, mice genetically engineered with a tissue specific fluorescent protein tagged CD63 protein (ExoBow) crossed with genetically engineered mice developing PDAC (KPC model) can establish a true time course development of the PMN from the time of tumor initiation. Authors who developed this model, demonstrated for example the presence of preferential communication routes through exosomes with CAFs and EC in PDAC mice and an increase in inter-organ communication with the kidneys, lungs, and thymus [133]. Endogenous models require engineered cells expressing a chimeric protein made of a fluorescent or luminescent protein and an EV-specific protein, and–based on our experience- the tracking of TEVs and isolation of TEV-capturing cells is more challenging due to their small numbers. Furthermore, these models do not distinguish the different EVs and other non-vesicular components of the secretome involved in the formation of the PMN.

A common limitation to both models is that they have been used to generate gain of function experiments in support of a sufficient role for TEVs in metastasis, leaving the question whether TEVs are necessary for metastasis unanswered. These latter experiments remain challenging as genetic or pharmacological approaches to inhibit or block the production and/or release of EVs and their capture have remained inadequate due to the substantial number of pathways involved in both the biogenesis and the capture of EVs *in vivo* and their heterogeneity [134, 135]. As our understanding of the mechanisms by which TEVs trigger the PMN and promote the MN continue to increase, it is anticipated that targetable mechanisms will be identified providing an opportunity to design novel approaches to inhibit or prevent metastasis, something that has not been successfully attempted so far and represent an important objective.

## Supplementary Information

Below is the link to the electronic supplementary material.

Supplementary Material 1

Supplementary Material 2

Supplementary Material 3

## Data Availability

No datasets were generated or analysed during the current study.

## References

[1] Paget S (1889) The distribution of secondary growths in cancer of the breast. 1889. Cancer Metastasis Rev 8:98–101 2673568

[2] Fidler IJ (1973) Selection of successive tumour lines for metastasis. Nat New Biol 242:148–149. 10.1038/newbio242148a0 4512654 10.1038/newbio242148a0

[3] Fidler IJ (2002) Critical determinants of metastasis. Semin Cancer Biol 12:89–96. 10.1006/scbi.2001.0416 12027580 10.1006/scbi.2001.0416

[4] Guise TA (2002) The vicious cycle of bone metastases. J Musculoskelet Neuronal Interact 2:570–572 15758398

[5] de la Mata J, Uy HL, Guise TA et al (1995) Interleukin-6 enhances hypercalcemia and bone resorption mediated by parathyroid hormone-related protein in vivo. J Clin Invest 95:2846–2852. 10.1172/JCI117990 7769125 10.1172/JCI117990 PMC295971

[6] Kaplan RN, Riba RD, Zacharoulis S et al (2005) VEGFR1-positive Haematopoietic bone marrow progenitors initiate the pre-metastatic niche. Nature 438:820–827. 10.1038/nature04186 16341007 10.1038/nature04186 PMC2945882

[7] Patras L, Shaashua L, Matei I, Lyden D (2023) Immune determinants of the pre-metastatic niche. Cancer Cell 41:546–572. 10.1016/j.ccell.2023.02.018 36917952 10.1016/j.ccell.2023.02.018 PMC10170403

[8] Gao D, Nolan DJ, Mellick AS et al (2008) Endothelial progenitor cells control the angiogenic switch in mouse lung metastasis. Science 319:195–198. 10.1126/science.1150224 18187653 10.1126/science.1150224

[9] Li R, Wen A, Lin J (2020) Pro-Inflammatory cytokines in the formation of the Pre-Metastatic niche. Cancers 12:3752. 10.3390/cancers12123752 33322216 10.3390/cancers12123752 PMC7764404

[10] Kaczanowska S, Beury DW, Gopalan V et al (2021) Genetically engineered myeloid cells rebalance the core immune suppression program in metastasis. Cell 184:2033–2052e21. 10.1016/j.cell.2021.02.048 33765443 10.1016/j.cell.2021.02.048 PMC8344805

[11] Giles AJ, Reid CM, Evans JD et al (2016) Activation of hematopoietic Stem/Progenitor cells promotes immunosuppression within the Pre-metastatic niche. Cancer Res 76:1335–1347. 10.1158/0008-5472.CAN-15-0204 26719537 10.1158/0008-5472.CAN-15-0204 PMC4794356

[12] Bojmar L, Zambirinis CP, Hernandez JM et al (2024) Multi-parametric atlas of the pre-metastatic liver for prediction of metastatic outcome in early-stage pancreatic cancer. Nat Med 30:2170–2180. 10.1038/s41591-024-03075-7 38942992 10.1038/s41591-024-03075-7 PMC11416063

[13] Kaplan RN, Rafii S, Lyden D (2006) Preparing the soil: the premetastatic niche. Cancer Res 66:11089–11093. 10.1158/0008-5472.CAN-06-2407 17145848 10.1158/0008-5472.CAN-06-2407 PMC2952469

[14] Peinado H, Alečković M, Lavotshkin S et al (2012) Melanoma exosomes educate bone marrow progenitor cells toward a pro-metastatic phenotype through MET. Nat Med 18:883–891. 10.1038/nm.2753 22635005 10.1038/nm.2753 PMC3645291

[15] Théry C, Witwer KW, Aikawa E et al (2018) Minimal information for studies of extracellular vesicles 2018 (MISEV2018): a position statement of the international society for extracellular vesicles and update of the MISEV2014 guidelines. J Extracell Vesicles 7:1535750. 10.1080/20013078.2018.1535750 30637094 10.1080/20013078.2018.1535750 PMC6322352

[16] Tkach M, Théry C (2016) Communication by extracellular vesicles: where we are and where we need to go. Cell 164:1226–1232. 10.1016/j.cell.2016.01.043 26967288 10.1016/j.cell.2016.01.043

[17] Welsh JA, Goberdhan DCI, O’Driscoll L et al (2024) Minimal information for studies of extracellular vesicles (MISEV2023): from basic to advanced approaches. J Extracell Vesicles 13:e12404. 10.1002/jev2.12404 38326288 10.1002/jev2.12404 PMC10850029

[18] Pucci F, Garris C, Lai CP et al (2016) SCS macrophages suppress melanoma by restricting tumor-derived vesicle-B cell interactions. Science 352:242–246. 10.1126/science.aaf1328 26989197 10.1126/science.aaf1328 PMC4960636

[19] Hood JL, San RS, Wickline SA (2011) Exosomes released by melanoma cells prepare Sentinel lymph nodes for tumor metastasis. Cancer Res 71:3792–3801. 10.1158/0008-5472.CAN-10-4455 21478294 10.1158/0008-5472.CAN-10-4455

[20] Hoshino A, Costa-Silva B, Shen T-L et al (2015) Tumour exosome integrins determine organotropic metastasis. Nature 527:329–335. 10.1038/nature15756 26524530 10.1038/nature15756 PMC4788391

[21] Linxweiler J, Kolbinger A, Himbert D et al (2021) Organ-Specific uptake of extracellular vesicles secreted by urological cancer cells. Cancers 13:4937. 10.3390/cancers13194937 34638418 10.3390/cancers13194937 PMC8508228

[22] Ghoroghi S, Mary B, Larnicol A et al (2021) Ral GTPases promote breast cancer metastasis by controlling biogenesis and organ targeting of exosomes. eLife 10:e61539. 10.7554/eLife.61539 33404012 10.7554/eLife.61539 PMC7822591

[23] Li S, Fu X, Ning D et al (2025) Colon cancer exosome-associated HSP90B1 initiates pre-metastatic niche formation in the liver by polarizing M1 macrophage into M2 phenotype. Biol Direct 20:52. 10.1186/s13062-025-00623-0 40234961 10.1186/s13062-025-00623-0 PMC12001560

[24] Qiu S, Xie L, Lu C et al (2022) Gastric cancer-derived Exosomal miR-519a-3p promotes liver metastasis by inducing intrahepatic M2-like macrophage-mediated angiogenesis. J Exp Clin Cancer Res CR 41:296. 10.1186/s13046-022-02499-8 36217165 10.1186/s13046-022-02499-8 PMC9549645

[25] Sun H, Meng Q, Shi C et al (2021) Hypoxia-Inducible exosomes facilitate liver-tropic premetastatic niche in colorectal cancer. Hepatol Baltim Md 74:2633–2651. 10.1002/hep.32009 10.1002/hep.32009 34110633

[26] Zhou J, Song Q, Li H et al (2024) Targeting circ-0034880-enriched tumor extracellular vesicles to impede SPP1highCD206 + pro-tumor macrophages mediated pre-metastatic niche formation in colorectal cancer liver metastasis. Mol Cancer 23:168. 10.1186/s12943-024-02086-9 39164758 10.1186/s12943-024-02086-9 PMC11334400

[27] Jiang K, Chen H, Fang Y et al (2021) Exosomal ANGPTL1 attenuates colorectal cancer liver metastasis by regulating Kupffer cell secretion pattern and impeding MMP9 induced vascular leakiness. J Exp Clin Cancer Res CR 40:21. 10.1186/s13046-020-01816-3 33413536 10.1186/s13046-020-01816-3 PMC7792106

[28] Gu J, Chu X, Huo Y et al (2023) Gastric cancer-derived exosomes facilitate pulmonary metastasis by activating ERK-mediated immunosuppressive macrophage polarization. J Cell Biochem 124:557–572. 10.1002/jcb.30390 36842167 10.1002/jcb.30390

[29] Morrissey SM, Zhang F, Ding C et al (2021) Tumor-derived exosomes drive immunosuppressive macrophages in a pre-metastatic niche through glycolytic dominant metabolic reprogramming. Cell Metab 33:2040–2058e10. 10.1016/j.cmet.2021.09.002 34559989 10.1016/j.cmet.2021.09.002 PMC8506837

[30] Zhao S, Mi Y, Guan B et al (2020) Tumor-derived Exosomal miR-934 induces macrophage M2 polarization to promote liver metastasis of colorectal cancer. J Hematol OncolJ Hematol Oncol 13:156. 10.1186/s13045-020-00991-2 33213490 10.1186/s13045-020-00991-2 PMC7678301

[31] Lu F, Ye M, Shen Y et al (2024) Hypoxic tumor-derived Exosomal miR-4488 induces macrophage M2 polarization to promote liver metastasis of pancreatic neuroendocrine neoplasm through RTN3/FABP5 mediated fatty acid oxidation. Int J Biol Sci 20:3201–3218. 10.7150/ijbs.96831 38904015 10.7150/ijbs.96831 PMC11186367

[32] Wu K, Li Y, Ji Y et al (2024) Tumor-Derived RAB21 + ABHD12+ sEVs drive the premetastatic microenvironment in the lung. Cancer Immunol Res 12:161–179. 10.1158/2326-6066.CIR-23-0221 38215051 10.1158/2326-6066.CIR-23-0221

[33] Zhong L, Liao D, Li J et al (2021) Rab22a-NeoF1 fusion protein promotes osteosarcoma lung metastasis through its secretion into exosomes. Signal Transduct Target Ther 6:59. 10.1038/s41392-020-00414-1 33568623 10.1038/s41392-020-00414-1 PMC7876000

[34] Zhang Z, Yao Z, Zhang Z et al (2023) Local radiotherapy for murine breast cancer increases risk of metastasis by promoting the recruitment of M-MDSCs in lung. Cancer Cell Int 23:107. 10.1186/s12935-023-02934-6 37268941 10.1186/s12935-023-02934-6 PMC10236833

[35] Zhang Y, Wang X, Gu Y et al (2025) Complement C3 of tumor-derived extracellular vesicles promotes metastasis of RCC via recruitment of immunosuppressive myeloid cells. Proc Natl Acad Sci U S A 122:e2420005122. 10.1073/pnas.2420005122 39847320 10.1073/pnas.2420005122 PMC11789090

[36] Shao Y, Chen T, Zheng X et al (2018) Colorectal cancer-derived small extracellular vesicles Establish an inflammatory premetastatic niche in liver metastasis. Carcinogenesis 39:1368–1379. 10.1093/carcin/bgy115 30184100 10.1093/carcin/bgy115

[37] Papiewska-Pająk I, Przygodzka P, Krzyżanowski D et al (2021) Snail overexpression alters the MicroRNA content of extracellular vesicles released from HT29 colorectal cancer cells and activates Pro-Inflammatory state in vivo. Cancers 13:172. 10.3390/cancers13020172 33419021 10.3390/cancers13020172 PMC7830966

[38] Fietta A, Fusco P, Germano G et al (2024) Neuroblastoma-derived hypoxic extracellular vesicles promote metastatic dissemination in a zebrafish model. PLoS ONE 19:e0316103. 10.1371/journal.pone.0316103 39715212 10.1371/journal.pone.0316103 PMC11666040

[39] Deng C, Xu Y, Chen H et al (2024) Extracellular-vesicle-packaged S100A11 from osteosarcoma cells mediates lung premetastatic niche formation by recruiting gMDSCs. Cell Rep 43:113751. 10.1016/j.celrep.2024.113751 38341855 10.1016/j.celrep.2024.113751

[40] Yin X, Tian M, Zhang J et al (2022) MiR-26b-5p in small extracellular vesicles derived from dying tumor cells after irradiation enhances the metastasis promoting microenvironment in esophageal squamous cell carcinoma. Cancer Lett 541:215746. 10.1016/j.canlet.2022.215746 35594995 10.1016/j.canlet.2022.215746

[41] Leary N, Walser S, He Y et al (2022) Melanoma-derived extracellular vesicles mediate lymphatic remodelling and impair tumour immunity in draining lymph nodes. J Extracell Vesicles 11:e12197. 10.1002/jev2.12197 35188342 10.1002/jev2.12197 PMC8859913

[42] Xu W, Patel N, Deng Y et al (2023) Extracellular vesicle-derived LINC00482 induces microglial M2 polarization to facilitate brain metastasis of NSCLC. Cancer Lett 561:216146. 10.1016/j.canlet.2023.216146 36963460 10.1016/j.canlet.2023.216146

[43] Li H, Zeng C, Shu C et al (2022) Laminins in tumor-derived exosomes upregulated by ETS1 reprogram omental macrophages to promote omental metastasis of ovarian cancer. Cell Death Dis 13:1028. 10.1038/s41419-022-05472-7 36477408 10.1038/s41419-022-05472-7 PMC9729302

[44] Horie M, Takagane K, Itoh G et al (2024) Exosomes secreted by ST3GAL5high cancer cells promote peritoneal dissemination by Establishing a premetastatic microenvironment. Mol Oncol 18:21–43. 10.1002/1878-0261.13524 37716915 10.1002/1878-0261.13524 PMC10766203

[45] Takano Y, Masuda T, Iinuma H et al (2017) Circulating Exosomal microRNA-203 is associated with metastasis possibly via inducing tumor-associated macrophages in colorectal cancer. Oncotarget 8:78598–78613. 10.18632/oncotarget.20009 29108252 10.18632/oncotarget.20009 PMC5667985

[46] Hyenne V, Ghoroghi S, Collot M et al (2019) Studying the fate of tumor extracellular vesicles at high Spatiotemporal resolution using the zebrafish embryo. Dev Cell 48:554–572e7. 10.1016/j.devcel.2019.01.014 30745140 10.1016/j.devcel.2019.01.014

[47] Plebanek MP, Angeloni NL, Vinokour E et al (2017) Pre-metastatic cancer exosomes induce immune surveillance by patrolling monocytes at the metastatic niche. Nat Commun 8:1319. 10.1038/s41467-017-01433-3 29105655 10.1038/s41467-017-01433-3 PMC5673063

[48] Schuldner M, Dörsam B, Shatnyeva O et al (2019) Exosome-dependent immune surveillance at the metastatic niche requires BAG6 and CBP/p300-dependent acetylation of p53. Theranostics 9:6047–6062. 10.7150/thno.36378 31534536 10.7150/thno.36378 PMC6735508

[49] Zhang C, Chen W, Pan S et al (2023) SEVs-mediated miR-6750 transfer inhibits pre-metastatic niche formation in nasopharyngeal carcinoma by targeting M6PR. Cell Death Discov 9:2. 10.1038/s41420-022-01262-4 10.1038/s41420-022-01262-4 PMC9823008 36609569

[50] Umakoshi M, Takahashi S, Itoh G et al (2019) Macrophage-mediated transfer of cancer-derived components to stromal cells contributes to establishment of a pro-tumor microenvironment. Oncogene 38:2162–2176. 10.1038/s41388-018-0564-x 30459356 10.1038/s41388-018-0564-x

[51] Mazumdar A, Urdinez J, Boro A et al (2020) Exploring the role of osteosarcoma-Derived extracellular vesicles in Pre-Metastatic niche formation and metastasis in the 143-B xenograft mouse osteosarcoma model. Cancers 12:3457. 10.3390/cancers12113457 33233625 10.3390/cancers12113457 PMC7699714

[52] Benito-Martin A, Nogués L, Hergueta-Redondo M et al (2023) Mast cells impair melanoma cell homing and metastasis by inhibiting HMGA1 secretion. Immunology 168:362–373. 10.1111/imm.13604 36352838 10.1111/imm.13604

[53] Zhang L, Pan J, Wang M et al (2025) Chronic Stress-Induced and tumor derived SP1 + Exosomes polarizing IL-1β + Neutrophils to increase lung metastasis of breast cancer. Adv Sci Weinh Baden-Wurtt Ger 12:e2310266. 10.1002/advs.202310266 10.1002/advs.202310266 PMC11789585 39630109

[54] Qi M, Xia Y, Wu Y et al (2022) Lin28B-high breast cancer cells promote immune suppression in the lung pre-metastatic niche via exosomes and support cancer progression. Nat Commun 13:897. 10.1038/s41467-022-28438-x 35173168 10.1038/s41467-022-28438-x PMC8850492

[55] Li T, Li T, Liang Y et al (2025) Colorectal cancer cells-derived Exosomal miR-188-3p promotes liver metastasis by creating a pre-metastatic niche via activation of hepatic stellate cells. J Transl Med 23:369. 10.1186/s12967-025-06334-4 40134019 10.1186/s12967-025-06334-4 PMC11938777

[56] Zhang C, Wang X-Y, Zhang P et al (2022) Cancer-derived exosomal HSPC111 promotes colorectal cancer liver metastasis by reprogramming lipid metabolism in cancer-associated fibroblasts. Cell Death Dis 13:57. 10.1038/s41419-022-04506-4 35027547 10.1038/s41419-022-04506-4 PMC8758774

[57] Dudgeon C, Casabianca A, Harris C et al (2023) Netrin-1 feedforward mechanism promotes pancreatic cancer liver metastasis via hepatic stellate cell activation, retinoid, and ELF3 signaling. Cell Rep 42:113369. 10.1016/j.celrep.2023.113369 37922311 10.1016/j.celrep.2023.113369 PMC12270551

[58] Li R, Zhou J, Wu X et al (2022) Jianpi Jiedu recipe inhibits colorectal cancer liver metastasis via regulating ITGBL1-rich extracellular vesicles mediated activation of cancer-associated fibroblasts. Phytomedicine Int J Phytother Phytopharm 100:154082. 10.1016/j.phymed.2022.154082 10.1016/j.phymed.2022.154082 35381565

[59] Ji Q, Zhou L, Sui H et al (2020) Primary tumors release ITGBL1-rich extracellular vesicles to promote distal metastatic tumor growth through fibroblast-niche formation. Nat Commun 11:1211. 10.1038/s41467-020-14869-x 32139701 10.1038/s41467-020-14869-x PMC7058049

[60] Fang T, Lv H, Lv G et al (2018) Tumor-derived Exosomal miR-1247-3p induces cancer-associated fibroblast activation to foster lung metastasis of liver cancer. Nat Commun 9:191. 10.1038/s41467-017-02583-0 29335551 10.1038/s41467-017-02583-0 PMC5768693

[61] González-Callejo P, Gener P, Díaz-Riascos ZV et al (2023) Extracellular vesicles secreted by triple-negative breast cancer stem cells trigger premetastatic niche remodeling and metastatic growth in the lungs. Int J Cancer 152:2153–2165. 10.1002/ijc.34447 36705298 10.1002/ijc.34447

[62] Du C, Duan X, Yao X et al (2020) Tumour-derived Exosomal miR-3473b promotes lung tumour cell intrapulmonary colonization by activating the nuclear factor-κB of local fibroblasts. J Cell Mol Med 24:7802–7813. 10.1111/jcmm.15411 32449597 10.1111/jcmm.15411 PMC7348150

[63] Jia W, Liang S, Lin W et al (2023) Hypoxia-induced exosomes facilitate lung pre-metastatic niche formation in hepatocellular carcinoma through the miR-4508-RFX1-IL17A-p38 MAPK-NF-κB pathway. Int J Biol Sci 19:4744–4762. 10.7150/ijbs.86767 37781522 10.7150/ijbs.86767 PMC10539707

[64] Chen Y, Pan G, Yang Y et al (2025) Tumor Exosomal RNPEP promotes lung metastasis of liver cancer via inducing cancer-associated fibroblast activation. Cancer Sci 116:792–807. 10.1111/cas.16417 39658708 10.1111/cas.16417 PMC11875778

[65] Tang T, Yang T, Xue H et al (2025) Breast cancer stem cell-derived Exosomal lnc-PDGFD induces fibroblast-niche formation and promotes lung metastasis. Oncogene 44:601–617. 10.1038/s41388-024-03237-4 39633064 10.1038/s41388-024-03237-4 PMC11850284

[66] Lin S, Shu L, Guo Y et al (2024) Cargo-eliminated osteosarcoma-derived small extracellular vesicles mediating competitive cellular uptake for inhibiting pulmonary metastasis of osteosarcoma. J Nanobiotechnol 22:360. 10.1186/s12951-024-02636-9 10.1186/s12951-024-02636-9 PMC11193292 38907233

[67] Sun X, Wang X, Yan C et al (2022) Tumor cell-released LC3-positive EVs promote lung metastasis of breast cancer through enhancing premetastatic niche formation. Cancer Sci 113:3405–3416. 10.1111/cas.15507 35879596 10.1111/cas.15507 PMC9530874

[68] Gener Lahav T, Adler O, Zait Y et al (2019) Melanoma-derived extracellular vesicles instigate Proinflammatory signaling in the metastatic microenvironment. Int J Cancer 145:2521–2534. 10.1002/ijc.32521 31216364 10.1002/ijc.32521

[69] Mao X, Tey SK, Yeung CLS et al (2020) Nidogen 1-Enriched extracellular vesicles facilitate extrahepatic metastasis of liver cancer by activating pulmonary fibroblasts to secrete tumor necrosis factor receptor 1. Adv Sci Weinh Baden-Wurtt Ger 7:2002157. 10.1002/advs.202002157 10.1002/advs.202002157 PMC7640351 33173740

[70] Gong L, Li G, Yi X et al (2024) Tumor-derived small extracellular vesicles facilitate omental metastasis of ovarian cancer by triggering activation of mesenchymal stem cells. Cell Commun Signal CCS 22:47. 10.1186/s12964-023-01413-9 38233863 10.1186/s12964-023-01413-9 PMC10795335

[71] Jia W, Liang S, Jin M et al (2024) Oleanolic acid inhibits hypoxic tumor-derived exosomes-induced premetastatic niche formation in hepatocellular carcinoma by targeting ERK1/2-NFκB signaling. Phytomedicine Int J Phytother Phytopharm 126:155208. 10.1016/j.phymed.2023.155208 10.1016/j.phymed.2023.155208 38387275

[72] Wang M, Qin Z, Wan J et al (2022) Tumor-derived exosomes drive pre-metastatic niche formation in lung via modulating CCL1 + fibroblast and CCR8 + Treg cell interactions. Cancer Immunol Immunother CII 71:2717–2730. 10.1007/s00262-022-03196-3 35428909 10.1007/s00262-022-03196-3 PMC10992578

[73] Rigg E, Wang J, Xue Z et al (2023) Inhibition of extracellular vesicle-derived miR-146a-5p decreases progression of melanoma brain metastasis via Notch pathway dysregulation in astrocytes. J Extracell Vesicles 12:e12363. 10.1002/jev2.12363 37759347 10.1002/jev2.12363 PMC10533779

[74] Huang G, Xu G, Cao Q et al (2025) Role of GPX3 + astrocytes in breast cancer brain metastasis activated by Circulating tumor cell exosomes. NPJ Precis Oncol 9:64. 10.1038/s41698-025-00833-9 40055530 10.1038/s41698-025-00833-9 PMC11889224

[75] Gu P, Sun M, Li L et al (2021) Breast Tumor-Derived Exosomal MicroRNA-200b-3p promotes specific organ metastasis through regulating CCL2 expression in lung epithelial cells. Front Cell Dev Biol 9:657158. 10.3389/fcell.2021.657158 34249913 10.3389/fcell.2021.657158 PMC8264457

[76] Zeng Z, Li Y, Pan Y et al (2018) Cancer-derived Exosomal miR-25-3p promotes pre-metastatic niche formation by inducing vascular permeability and angiogenesis. Nat Commun 9:5395. 10.1038/s41467-018-07810-w 30568162 10.1038/s41467-018-07810-w PMC6300604

[77] Yao Y, Qian R, Gao H et al (2024) LSD1 deficiency in breast cancer cells promotes the formation of pre-metastatic niches. NPJ Precis Oncol 8:260. 10.1038/s41698-024-00751-2 39528717 10.1038/s41698-024-00751-2 PMC11555121

[78] Yokota Y, Noda T, Okumura Y et al (2021) Serum Exosomal miR-638 is a prognostic marker of HCC via downregulation of VE-cadherin and ZO-1 of endothelial cells. Cancer Sci 112:1275–1288. 10.1111/cas.14807 33426736 10.1111/cas.14807 PMC7935782

[79] Jin J, Cui Y, Niu H et al (2024) NSCLC extracellular vesicles containing miR-374a-5p promote leptomeningeal metastasis by influencing Blood–Brain barrier permeability. Mol Cancer Res MCR 22:699–710. 10.1158/1541-7786.MCR-24-0052 38639925 10.1158/1541-7786.MCR-24-0052 PMC11294816

[80] Mao Y, Wang J, Wang Y et al (2024) Hypoxia induced Exosomal Circ-ZNF609 promotes pre-metastatic niche formation and cancer progression via miR-150-5p/VEGFA and HuR/ZO-1 axes in esophageal squamous cell carcinoma. Cell Death Discov 10:133. 10.1038/s41420-024-01905-8 38472174 10.1038/s41420-024-01905-8 PMC10933275

[81] Xie L, Zhang K, You B et al (2023) Hypoxic nasopharyngeal carcinoma-derived Exosomal miR-455 increases vascular permeability by targeting ZO-1 to promote metastasis. Mol Carcinog 62:803–819. 10.1002/mc.23525 36929868 10.1002/mc.23525

[82] Duan S, Nordmeier S, Byrnes AE, Buxton ILO (2021) Extracellular Vesicle-Mediated purinergic signaling contributes to host microenvironment plasticity and metastasis in triple negative breast cancer. Int J Mol Sci 22:597. 10.3390/ijms22020597 33435297 10.3390/ijms22020597 PMC7827112

[83] Yang W-W, Yang L-Q, Zhao F et al (2017) Epiregulin promotes lung metastasis of salivary adenoid cystic carcinoma. Theranostics 7:3700–3714. 10.7150/thno.19712 29109770 10.7150/thno.19712 PMC5667342

[84] Kimoto A, Kadoi Y, Tsuruda T et al (2023) Exosomes in Ascites from patients with human pancreatic cancer enhance remote metastasis partially through endothelial-mesenchymal transition. Pancreatol Off J Int Assoc Pancreatol IAP Al 23:377–388. 10.1016/j.pan.2023.04.002 10.1016/j.pan.2023.04.002 37088585

[85] Li K, Xue W, Lu Z et al (2024) Tumor-derived Exosomal ADAM17 promotes pre-metastatic niche formation by enhancing vascular permeability in colorectal cancer. J Exp Clin Cancer Res CR 43:59. 10.1186/s13046-024-02991-3 38413999 10.1186/s13046-024-02991-3 PMC10898123

[86] Dong Q, Dong M, Liu X et al (2024) Salivary adenoid cystic carcinoma-derived α2,6-sialylated extracellular vesicles increase vascular permeability by triggering ER-stress in endothelial cells and promote lung metastasis. Cancer Lett 611:217407. 10.1016/j.canlet.2024.217407 39710056 10.1016/j.canlet.2024.217407

[87] Ma Z, Wei K, Yang F et al (2021) Tumor-derived Exosomal miR-3157-3p promotes angiogenesis, vascular permeability and metastasis by targeting TIMP/KLF2 in non-small cell lung cancer. Cell Death Dis 12:840. 10.1038/s41419-021-04037-4 34497265 10.1038/s41419-021-04037-4 PMC8426367

[88] Biagioni A, Laurenzana A, Menicacci B et al (2021) uPAR-expressing melanoma exosomes promote angiogenesis by VE-Cadherin, EGFR and uPAR overexpression and rise of ERK1,2 signaling in endothelial cells. Cell Mol Life Sci CMLS 78:3057–3072. 10.1007/s00018-020-03707-4 33237352 10.1007/s00018-020-03707-4 PMC8004497

[89] Keklikoglou I, Cianciaruso C, Güç E et al (2019) Chemotherapy elicits pro-metastatic extracellular vesicles in breast cancer models. Nat Cell Biol 21:190–202. 10.1038/s41556-018-0256-3 30598531 10.1038/s41556-018-0256-3 PMC6525097

[90] Heo W, Lee W, Cheun JH et al (2023) Triple-Negative breast Cancer-Derived extracellular vesicles promote a hepatic premetastatic niche via a cascade of microenvironment remodeling. Mol Cancer Res MCR 21:726–740. 10.1158/1541-7786.MCR-22-0673 37040163 10.1158/1541-7786.MCR-22-0673

[91] Zheng X, Lu S, He Z et al (2020) MCU-dependent negative sorting of miR-4488 to extracellular vesicles enhances angiogenesis and promotes breast cancer metastatic colonization. Oncogene 39:6975–6989. 10.1038/s41388-020-01514-6 33067576 10.1038/s41388-020-01514-6

[92] Busatto S, Song T-H, Kim HJ et al (2025) Breast Cancer-Derived extracellular vesicles modulate the cytoplasmic and cytoskeletal dynamics of Blood-Brain barrier endothelial cells. J Extracell Vesicles 14:e70038. 10.1002/jev2.70038 39868462 10.1002/jev2.70038 PMC11770372

[93] Gan D-X, Wang Y-B, He M-Y et al (2020) Lung cancer Cells-Controlled Dkk-1 production in brain metastatic cascade drive microglia to acquirea Pro-tumorigenic phenotype. Front Cell Dev Biol 8:591405. 10.3389/fcell.2020.591405 33384994 10.3389/fcell.2020.591405 PMC7769850

[94] Mary B, Asokan N, Jerabkova-Roda K et al (2023) Blood flow diverts extracellular vesicles from endothelial degradative compartments to promote angiogenesis. EMBO Rep 24:e57042. 10.15252/embr.202357042 37971863 10.15252/embr.202357042 PMC10702841

[95] Xu J, Wang H, Shi B et al (2023) Exosomal MFI2-AS1 sponge miR-107 promotes non-small cell lung cancer progression through NFAT5. Cancer Cell Int 23:51. 10.1186/s12935-023-02886-x 36934264 10.1186/s12935-023-02886-x PMC10024841

[96] Lin Y, Zheng H, Jia L et al (2024) Integrin α6-containing extracellular vesicles promote lymphatic remodelling for pre-metastatic niche formation in lymph nodes via interplay with CD151. J Extracell Vesicles 13:e12518. 10.1002/jev2.12518 39329462 10.1002/jev2.12518 PMC11428163

[97] Liu D, Li C, Trojanowicz B et al (2016) CD97 promotion of gastric carcinoma lymphatic metastasis is exosome dependent. Gastric Cancer Off J Int Gastric Cancer Assoc Jpn Gastric Cancer Assoc 19:754–766. 10.1007/s10120-015-0523-y 10.1007/s10120-015-0523-y PMC4906076 26233326

[98] García-Silva S, Benito-Martín A, Nogués L et al (2021) Melanoma-derived small extracellular vesicles induce lymphangiogenesis and metastasis through an NGFR-dependent mechanism. Nat Cancer 2:1387–1405. 10.1038/s43018-021-00272-y 34957415 10.1038/s43018-021-00272-y PMC8697753

[99] Han N, Zhou D, Ruan M et al (2023) Cancer cell-derived extracellular vesicles drive pre-metastatic niche formation of lymph node via IFNGR1/JAK1/STAT1-activated-PD-L1 expression on FRCs in head and neck cancer. Oral Oncol 145:106524. 10.1016/j.oraloncology.2023.106524 37482043 10.1016/j.oraloncology.2023.106524

[100] Su X, Brassard A, Bartolomucci A et al (2023) Tumour extracellular vesicles induce neutrophil extracellular traps to promote lymph node metastasis. J Extracell Vesicles 12:e12341. 10.1002/jev2.12341 37563798 10.1002/jev2.12341 PMC10415595

[101] Sun B, Zhou Y, Fang Y et al (2019) Colorectal cancer exosomes induce lymphatic network remodeling in lymph nodes. Int J Cancer 145:1648–1659. 10.1002/ijc.32196 30734278 10.1002/ijc.32196

[102] Henrich SE, McMahon KM, Plebanek MP et al (2020) Prostate cancer extracellular vesicles mediate intercellular communication with bone marrow cells and promote metastasis in a cholesterol-dependent manner. J Extracell Vesicles 10:e12042. 10.1002/jev2.12042 33408816 10.1002/jev2.12042 PMC7775568

[103] Ma Q, Liang M, Wu Y et al (2021) Small extracellular vesicles deliver osteolytic effectors and mediate cancer-induced osteolysis in bone metastatic niche. J Extracell Vesicles 10:e12068. 10.1002/jev2.12068 33659051 10.1002/jev2.12068 PMC7892803

[104] Yuan X, Qian N, Ling S et al (2021) Breast cancer exosomes contribute to pre-metastatic niche formation and promote bone metastasis of tumor cells. Theranostics 11:1429–1445. 10.7150/thno.45351 33391543 10.7150/thno.45351 PMC7738874

[105] Zhang S, Liao X, Chen S et al (2022) Large Oncosome-Loaded VAPA promotes bone-tropic metastasis of hepatocellular carcinoma via formation of osteoclastic Pre-Metastatic niche. Adv Sci Weinh Baden-Wurtt Ger 9:e2201974. 10.1002/advs.202201974 10.1002/advs.202201974 PMC9631052 36169100

[106] Qiu R, Deng Y, Lu Y et al (2024) ITGB3-enriched extracellular vesicles mediate the formation of osteoclastic pre-metastatic niche to promote lung adenocarcinoma bone metastasis. Mol Carcinog 63:2190–2204. 10.1002/mc.23803 39136603 10.1002/mc.23803

[107] Wang J, Du X, Wang X et al (2022) Tumor-derived miR-378a-3p-containing extracellular vesicles promote osteolysis by activating the Dyrk1a/Nfatc1/Angptl2 axis for bone metastasis. Cancer Lett 526:76–90. 10.1016/j.canlet.2021.11.017 34801597 10.1016/j.canlet.2021.11.017

[108] Wei X, Liang M, Deng M et al (2024) A switch from lysosomal degradation to secretory autophagy initiates osteogenic bone metastasis in prostate cancer. J Extracell Vesicles13:e70002. 10.1002/jev2.70002 39497621 10.1002/jev2.70002 PMC11535520

[109] Brown TJ, Rutland CS, Choi KK et al (2024) Modulation of the pre-metastatic bone niche: molecular changes mediated by bone-homing prostate cancer extracellular vesicles. Front Cell Dev Biol 12:1354606. 10.3389/fcell.2024.1354606 38455075 10.3389/fcell.2024.1354606 PMC10919403

[110] Furesi G, de Jesus Domingues AM, Alexopoulou D et al (2022) Exosomal MiRNAs from prostate cancer impair osteoblast function in mice. Int J Mol Sci 23:1285. 10.3390/ijms23031285 35163219 10.3390/ijms23031285 PMC8836054

[111] Li X-Q, Zhang R, Lu H et al (2022) Extracellular vesicle-packaged CDH11 and ITGA5 induce the premetastatic niche for bone colonization of breast cancer cells. Cancer Res 82:1560–1574. 10.1158/0008-5472.CAN-21-1331 35149589 10.1158/0008-5472.CAN-21-1331

[112] Xu J, Feng X, Yin N et al (2022) Exosomes from cisplatin-induced dormant cancer cells facilitate the formation of premetastatic niche in bone marrow through activating Glycolysis of BMSCs. Front Oncol 12:922465. 10.3389/fonc.2022.922465 36568212 10.3389/fonc.2022.922465 PMC9786109

[113] Ghoshal A, Rodrigues LC, Gowda CP et al (2019) Extracellular vesicle-dependent effect of RNA-binding protein IGF2BP1 on melanoma metastasis. Oncogene 38:4182–4196. 10.1038/s41388-019-0797-3 30936459 10.1038/s41388-019-0797-3 PMC7727312

[114] Wang Y, Li Y, Zhong J et al (2023) Tumor-derived Cav-1 promotes pre-metastatic niche formation and lung metastasis in breast cancer. Theranostics 13:1684–1697. 10.7150/thno.79250 37056561 10.7150/thno.79250 PMC10086203

[115] Zhu G, Wang L, Meng W et al (2021) LOXL2-enriched small extracellular vesicles mediate hypoxia-induced premetastatic niche and indicates poor outcome of head and neck cancer. Theranostics 11:9198–9216. 10.7150/thno.62455 34646366 10.7150/thno.62455 PMC8490529

[116] Yan Y, Du C, Duan X et al (2022) Inhibiting collagen I production and tumor cell colonization in the lung via miR-29a-3p loading of exosome-/liposome-based nanovesicles. Acta Pharm Sin B 12:939–951. 10.1016/j.apsb.2021.08.011 10.1016/j.apsb.2021.08.011 PMC8897025 35256956

[117] Costa-Silva B, Aiello NM, Ocean AJ et al (2015) Pancreatic cancer exosomes initiate pre-metastatic niche formation in the liver. Nat Cell Biol 17:816–826. 10.1038/ncb3169 25985394 10.1038/ncb3169 PMC5769922

[118] Shinde A, Paez JS, Libring S et al (2020) Transglutaminase-2 facilitates extracellular vesicle-mediated establishment of the metastatic niche. Oncogenesis 9:16. 10.1038/s41389-020-0204-5 32054828 10.1038/s41389-020-0204-5 PMC7018754

[119] Rana S, Malinowska K, Zöller M (2013) Exosomal tumor MicroRNA modulates premetastatic organ cells. Neoplasia N Y N 15:281–295. 10.1593/neo.122010 10.1593/neo.122010 PMC3593151 23479506

[120] Deep G, Jain A, Kumar A et al (2020) Exosomes secreted by prostate cancer cells under hypoxia promote matrix metalloproteinases activity at pre-metastatic niches. Mol Carcinog 59:323–332. 10.1002/mc.23157 31943365 10.1002/mc.23157 PMC7189745

[121] Macklin R, Wang H, Loo D et al (2016) Extracellular vesicles secreted by highly metastatic clonal variants of osteosarcoma preferentially localize to the lungs and induce metastatic behaviour in poorly metastatic clones. Oncotarget 7:43570–43587. 10.18632/oncotarget.9781 27259278 10.18632/oncotarget.9781 PMC5190045

[122] Sun J, Lu Z, Fu W et al (2021) Exosome-Derived ADAM17 promotes liver metastasis in colorectal cancer. Front Pharmacol 12:734351. 10.3389/fphar.2021.734351 34650435 10.3389/fphar.2021.734351 PMC8506248

[123] Verweij FJ, Revenu C, Arras G et al (2019) Live tracking of Inter-organ communication by endogenous exosomes. Vivo Dev Cell 48:573–589e4. 10.1016/j.devcel.2019.01.004 30745143 10.1016/j.devcel.2019.01.004

[124] Zomer A, Maynard C, Verweij FJ et al (2015) In vivo imaging reveals extracellular vesicle-mediated phenocopying of metastatic behavior. Cell 161:1046–1057. 10.1016/j.cell.2015.04.042 26000481 10.1016/j.cell.2015.04.042 PMC4448148

[125] Androuin A, Verweij FJ, van Niel G (2021) Zebrafish as a preclinical model for extracellular vesicle-based therapeutic development. Adv Drug Deliv Rev 176:113815. 10.1016/j.addr.2021.05.025 34058284 10.1016/j.addr.2021.05.025

[126] Verweij FJ, Balaj L, Boulanger CM et al (2021) The power of imaging to understand extracellular vesicle biology in vivo. Nat Methods 18:1013–1026. 10.1038/s41592-021-01206-3 34446922 10.1038/s41592-021-01206-3 PMC8796660

[127] Dai J, Escara-Wilke J, Keller JM et al (2019) Primary prostate cancer educates bone stroma through Exosomal pyruvate kinase M2 to promote bone metastasis. J Exp Med 216:2883–2899. 10.1084/jem.20190158 31548301 10.1084/jem.20190158 PMC6888980

[128] Blavier L, Nakata R, Neviani P et al (2023) The capture of extracellular vesicles endogenously released by xenotransplanted tumours induces an inflammatory reaction in the premetastatic niche. J Extracell Vesicles 12:e12326. 10.1002/jev2.12326 37194998 10.1002/jev2.12326 PMC10190125

[129] Ortiz A, Gui J, Zahedi F et al (2019) An interferon-driven oxysterol-based defense against tumor-derived extracellular vesicles. Cancer Cell 35:33–45e6. 10.1016/j.ccell.2018.12.001 30645975 10.1016/j.ccell.2018.12.001 PMC6336114

[130] Choi H, Lee DS (2016) Illuminating the physiology of extracellular vesicles. Stem Cell Res Ther 7:55. 10.1186/s13287-016-0316-1 27084088 10.1186/s13287-016-0316-1 PMC4833943

[131] Pužar Dominkuš P, Stenovec M, Sitar S et al (2018) PKH26 labeling of extracellular vesicles: characterization and cellular internalization of contaminating PKH26 nanoparticles. Biochim Biophys Acta Biomembr 1860:1350–1361. 10.1016/j.bbamem.2018.03.013 29551275 10.1016/j.bbamem.2018.03.013

[132] van Niel G, Carter DRF, Clayton A et al (2022) Challenges and directions in studying cell-cell communication by extracellular vesicles. Nat Rev Mol Cell Biol 23:369–382. 10.1038/s41580-022-00460-3 35260831 10.1038/s41580-022-00460-3

[133] Adem B, Bastos N, Ruivo CF et al (2024) Exosomes define a local and systemic communication network in healthy pancreas and pancreatic ductal adenocarcinoma. Nat Commun 15:1496. 10.1038/s41467-024-45753-7 38383468 10.1038/s41467-024-45753-7 PMC10881969

[134] Cocozza F, Grisard E, Martin-Jaular L et al (2020) SnapShot: extracellular vesicles. Cell 182:262–262. 10.1016/j.cell.2020.04.054. .e1 32649878 10.1016/j.cell.2020.04.054

[135] Mathieu M, Martin-Jaular L, Lavieu G, Théry C (2019) Specificities of secretion and uptake of exosomes and other extracellular vesicles for cell-to-cell communication. Nat Cell Biol 21:9–17. 10.1038/s41556-018-0250-9 30602770 10.1038/s41556-018-0250-9

