# Supplementary material for: Contribution of tumor-derived extracellular vesicles in the establishment of the pre-metastatic niche: lessons learned from past experimentations and future directions
Source: Clin Exp Metastasis. 2026 Mar 7;43(2):16. doi: 10.1007/s10585-026-10396-z (PMC12967406; doi:10.1007/s10585-026-10396-z)
Supplement: Supplementary file 1 — Supplementary Material 1 [file 10585_2026_10396_MOESM1_ESM.docx]

| **PMID** | **First Author** | **Title** | **Journal/Book** | **Publication Year** |
| --- | --- | --- | --- | --- |
| 21478294 | Hood JL | Exosomes released by melanoma cells prepare sentinel lymph nodes for tumor metastasis | Cancer Res | 2011 |
| 22635005 | Peinado H | Melanoma exosomes educate bone marrow progenitor cells toward a pro-metastatic phenotype through MET | Nat Med | 2012 |
| 23479506 | Rana S | Exosomal tumor microRNA modulates premetastatic organ cells | Neoplasia | 2013 |
| 25985394 | Costa-Silva B | Pancreatic cancer exosomes initiate pre-metastatic niche formation in the liver | Nat Cell Biol | 2015 |
| 26000481 | Zomer A | In Vivo imaging reveals extracellular vesicle-mediated phenocopying of metastatic behavior | Cell | 2015 |
| 26233326 | Liu D | CD97 promotion of gastric carcinoma lymphatic metastasis is exosome dependent | Gastric Cancer | 2016 |
| 26524530 | Hoshino A | Tumour exosome integrins determine organotropic metastasis | Nature | 2015 |
| 26989197 | Pucci F | SCS macrophages suppress melanoma by restricting tumor-derived vesicle-B cell interactions | Science | 2016 |
| 27259278 | Macklin R | Extracellular vesicles secreted by highly metastatic clonal variants of osteosarcoma preferentially localize to the lungs and induce metastatic behaviour in poorly metastatic clones | Oncotarget | 2016 |
| 28969005 | Yu Z | Pancreatic cancer-derived exosomes promote tumor metastasis and liver pre-metastatic niche formation | Oncotarget | 2017 |
| 29105655 | Plebanek MP | Pre-metastatic cancer exosomes induce immune surveillance by patrolling monocytes at the metastatic niche | Nat Commun | 2017 |
| 29108252 | Takano Y | Circulating exosomal microRNA-203 is associated with metastasis possibly via inducing tumor-associated macrophages in colorectal cancer | Oncotarget | 2017 |
| 29109770 | Yang WW | Epiregulin Promotes Lung Metastasis of Salivary Adenoid Cystic Carcinoma | Theranostics | 2017 |
| 29335551 | Fang T | Tumor-derived exosomal miR-1247-3p induces cancer-associated fibroblast activation to foster lung metastasis of liver cancer | Nat Commun | 2018 |
| 30184100 | Shao Y | Colorectal cancer-derived small extracellular vesicles establish an inflammatory premetastatic niche in liver metastasis | Carcinogenesis | 2018 |
| 30459356 | Umakoshi M | Macrophage-mediated transfer of cancer-derived components to stromal cells contributes to establishment of a pro-tumor microenvironment | Oncogene | 2019 |
| 30568162 | Zeng Z | Cancer-derived exosomal miR-25-3p promotes pre-metastatic niche formation by inducing vascular permeability and angiogenesis | Nat Commun | 2018 |
| 30598531 | Keklikoglou I | Chemotherapy elicits pro-metastatic extracellular vesicles in breast cancer models | Nat Cell Biol | 2019 |
| 30645975 | Ortiz A | An Interferon-Driven Oxysterol-Based Defense against Tumor-Derived Extracellular Vesicles | Cancer Cell | 2019 |
| 30734278 | Sun B | Colorectal cancer exosomes induce lymphatic network remodeling in lymph nodes | Int J Cancer | 2019 |
| 30745140 | Hyenne V | Studying the Fate of Tumor Extracellular Vesicles at High Spatiotemporal Resolution Using the Zebrafish Embryo | Dev Cell | 2019 |
| 30936459 | Ghoshal A | Extracellular vesicle-dependent effect of RNA-binding protein IGF2BP1 on melanoma metastasis | Oncogene | 2019 |
| 31216364 | Gener Lahav T | Melanoma-derived extracellular vesicles instigate proinflammatory signaling in the metastatic microenvironment | Int J Cancer | 2019 |
| 31534536 | Schuldner M | Exosome-dependent immune surveillance at the metastatic niche requires BAG6 and CBP/p300-dependent acetylation of p53 | Theranostics | 2019 |
| 31548301 | Dai J | Primary prostate cancer educates bone stroma through exosomal pyruvate kinase M2 to promote bone metastasis | J Exp Med | 2019 |
| 31943365 | Deep G | Exosomes secreted by prostate cancer cells under hypoxia promote matrix metalloproteinases activity at pre-metastatic niches | Mol Carcinog | 2020 |
| 32054828 | Shinde A | Transglutaminase-2 facilitates extracellular vesicle-mediated establishment of the metastatic niche | Oncogenesis | 2020 |
| 32139701 | Ji Q | Primary tumors release ITGBL1-rich extracellular vesicles to promote distal metastatic tumor growth through fibroblast-niche formation | Nat Commun | 2020 |
| 32449597 | Du C | Tumour-derived exosomal miR-3473b promotes lung tumour cell intrapulmonary colonization by activating the nuclear factor-κB of local fibroblasts | J Cell Mol Med | 2020 |
| 32901082 | Liu J | Immune suppressed tumor microenvironment by exosomes derived from gastric cancer cells via modulating immune functions | Sci Rep | 2020 |
| 33067576 | Zheng X | MCU-dependent negative sorting of miR-4488 to extracellular vesicles enhances angiogenesis and promotes breast cancer metastatic colonization | Oncogene | 2020 |
| 33115808 | Wills CA | Chemotherapy-Induced Upregulation of Small Extracellular Vesicle-Associated PTX3 Accelerates Breast Cancer Metastasis | Cancer Res | 2021 |
| 33173740 | Mao X | Nidogen 1-Enriched Extracellular Vesicles Facilitate Extrahepatic Metastasis of Liver Cancer by Activating Pulmonary Fibroblasts to Secrete Tumor Necrosis Factor Receptor 1 | Adv Sci (Weinh) | 2020 |
| 33213490 | Zhao S | Tumor-derived exosomal miR-934 induces macrophage M2 polarization to promote liver metastasis of colorectal cancer | J Hematol Oncol | 2020 |
| 33233625 | Mazumdar A | Exploring the Role of Osteosarcoma-Derived Extracellular Vesicles in Pre-Metastatic Niche Formation and Metastasis in the 143-B Xenograft Mouse Osteosarcoma Model | Cancers (Basel) | 2020 |
| 33237352 | Biagioni A | uPAR-expressing melanoma exosomes promote angiogenesis by VE-Cadherin, EGFR and uPAR overexpression and rise of ERK1,2 signaling in endothelial cells | Cell Mol Life Sci | 2021 |
| 33384994 | Gan DX | Lung Cancer Cells-Controlled Dkk-1 Production in Brain Metastatic Cascade Drive Microglia to Acquire a Pro-tumorigenic Phenotype | Front Cell Dev Biol | 2020 |
| 33391543 | Yuan X | Breast cancer exosomes contribute to pre-metastatic niche formation and promote bone metastasis of tumor cells | Theranostics | 2021 |
| 33404012 | Ghoroghi S | Ral GTPases promote breast cancer metastasis by controlling biogenesis and organ targeting of exosomes | Elife | 2021 |
| 33408816 | Henrich SE | Prostate cancer extracellular vesicles mediate intercellular communication with bone marrow cells and promote metastasis in a cholesterol-dependent manner | J Extracell Vesicles | 2020 |
| 33413536 | Jiang K | Exosomal ANGPTL1 attenuates colorectal cancer liver metastasis by regulating Kupffer cell secretion pattern and impeding MMP9 induced vascular leakiness | J Exp Clin Cancer Res | 2021 |
| 33419021 | Papiewska-Pająk I | Snail Overexpression Alters the microRNA Content of Extracellular Vesicles Released from HT29 Colorectal Cancer Cells and Activates Pro-Inflammatory State In Vivo | Cancers (Basel) | 2021 |
| 33426736 | Yokota Y | Serum exosomal miR-638 is a prognostic marker of HCC via downregulation of VE-cadherin and ZO-1 of endothelial cells | Cancer Sci | 2021 |
| 33435297 | Duan S | Extracellular Vesicle-Mediated Purinergic Signaling Contributes to Host Microenvironment Plasticity and Metastasis in Triple Negative Breast Cancer | Int J Mol Sci | 2021 |
| 33568623 | Zhong L | Rab22a-NeoF1 fusion protein promotes osteosarcoma lung metastasis through its secretion into exosomes | Signal Transduct Target Ther | 2021 |
| 33659051 | Ma Q | Small extracellular vesicles deliver osteolytic effectors and mediate cancer-induced osteolysis in bone metastatic niche | J Extracell Vesicles | 2021 |
| 34110633 | Sun H | Hypoxia-Inducible Exosomes Facilitate Liver-Tropic Premetastatic Niche in Colorectal Cancer | Hepatology | 2021 |
| 34249913 | Gu P | Breast Tumor-Derived Exosomal MicroRNA-200b-3p Promotes Specific Organ Metastasis Through Regulating CCL2 Expression in Lung Epithelial Cells | Front Cell Dev Biol | 2021 |
| 34497265 | Ma Z | Tumor-derived exosomal miR-3157-3p promotes angiogenesis, vascular permeability and metastasis by targeting TIMP/KLF2 in non-small cell lung cancer | Cell Death Dis | 2021 |
| 34559989 | Morrissey SM | Tumor-derived exosomes drive immunosuppressive macrophages in a pre-metastatic niche through glycolytic dominant metabolic reprogramming | Cell Metab | 2021 |
| 34638418 | Linxweiler J | Organ-Specific Uptake of Extracellular Vesicles Secreted by Urological Cancer Cells | Cancers (Basel) | 2021 |
| 34646366 | Zhu G | LOXL2-enriched small extracellular vesicles mediate hypoxia-induced premetastatic niche and indicates poor outcome of head and neck cancer | Theranostics | 2021 |
| 34650435 | Sun J | Exosome-Derived ADAM17 Promotes Liver Metastasis in Colorectal Cancer | Front Pharmacol | 2021 |
| 34801597 | Wang J | Tumor-derived miR-378a-3p-containing extracellular vesicles promote osteolysis by activating the Dyrk1a/Nfatc1/Angptl2 axis for bone metastasis | Cancer Lett | 2022 |
| 34957415 | García-Silva S | Melanoma-derived small extracellular vesicles induce lymphangiogenesis and metastasis through an NGFR-dependent mechanism | Nat Cancer | 2021 |
| 34958869 | Zhu M | Integration of exosomal miR-106a and mesothelial cells facilitates gastric cancer peritoneal dissemination | Cell Signal | 2022 |
| 35027547 | Zhang C | Cancer-derived exosomal HSPC111 promotes colorectal cancer liver metastasis by reprogramming lipid metabolism in cancer-associated fibroblasts | Cell Death Dis | 2022 |
| 35149589 | Li XQ | Extracellular Vesicle-Packaged CDH11 and ITGA5 Induce the Premetastatic Niche for Bone Colonization of Breast Cancer Cells | Cancer Res | 2022 |
| 35163219 | Furesi G | Exosomal miRNAs from Prostate Cancer Impair Osteoblast Function in Mice | Int J Mol Sci | 2022 |
| 35173168 | Qi M | Lin28B-high breast cancer cells promote immune suppression in the lung pre-metastatic niche via exosomes and support cancer progression | Nat Commun | 2022 |
| 35188342 | Leary N | Melanoma-derived extracellular vesicles mediate lymphatic remodelling and impair tumour immunity in draining lymph nodes | J Extracell Vesicles | 2022 |
| 35256956 | Yan Y | Inhibiting collagen I production and tumor cell colonization in the lung via miR-29a-3p loading of exosome-/liposome-based nanovesicles | Acta Pharm Sin B | 2022 |
| 35381565 | Li R | Jianpi Jiedu Recipe inhibits colorectal cancer liver metastasis via regulating ITGBL1-rich extracellular vesicles mediated activation of cancer-associated fibroblasts | Phytomedicine | 2022 |
| 35428909 | Wang M | Tumor-derived exosomes drive pre-metastatic niche formation in lung via modulating CCL1(+) fibroblast and CCR8(+) Treg cell interactions | Cancer Immunol Immunother | 2022 |
| 35594995 | Yin X | MiR-26b-5p in small extracellular vesicles derived from dying tumor cells after irradiation enhances the metastasis promoting microenvironment in esophageal squamous cell carcinoma | Cancer Lett | 2022 |
| 35879596 | Sun X | Tumor cell-released LC3-positive EVs promote lung metastasis of breast cancer through enhancing premetastatic niche formation | Cancer Sci | 2022 |
| 36169100 | Zhang S | Large Oncosome-Loaded VAPA Promotes Bone-Tropic Metastasis of Hepatocellular Carcinoma Via Formation of Osteoclastic Pre-Metastatic Niche | Adv Sci (Weinh) | 2022 |
| 36217165 | Qiu S | Gastric cancer-derived exosomal miR-519a-3p promotes liver metastasis by inducing intrahepatic M2-like macrophage-mediated angiogenesis | J Exp Clin Cancer Res | 2022 |
| 36352838 | Benito-Martin A | Mast cells impair melanoma cell homing and metastasis by inhibiting HMGA1 secretion | Immunology | 2023 |
| 36477408 | Li H | Laminins in tumor-derived exosomes upregulated by ETS1 reprogram omental macrophages to promote omental metastasis of ovarian cancer | Cell Death Dis | 2022 |
| 36568212 | Xu J | Exosomes from cisplatin-induced dormant cancer cells facilitate the formation of premetastatic niche in bone marrow through activating glycolysis of BMSCs | Front Oncol | 2022 |
| 36609569 | Zhang C | SEVs-mediated miR-6750 transfer inhibits pre-metastatic niche formation in nasopharyngeal carcinoma by targeting M6PR | Cell Death Discov | 2023 |
| 36705298 | González-Callejo P | Extracellular vesicles secreted by triple-negative breast cancer stem cells trigger premetastatic niche remodeling and metastatic growth in the lungs | Int J Cancer | 2023 |
| 36842167 | Gu J | Gastric cancer-derived exosomes facilitate pulmonary metastasis by activating ERK-mediated immunosuppressive macrophage polarization | J Cell Biochem | 2023 |
| 36929868 | Xie L | Hypoxic nasopharyngeal carcinoma-derived exosomal miR-455 increases vascular permeability by targeting ZO-1 to promote metastasis | Mol Carcinog | 2023 |
| 36934264 | Xu J | Exosomal MFI2-AS1 sponge miR-107 promotes non-small cell lung cancer progression through NFAT5 | Cancer Cell Int | 2023 |
| 36963460 | Xu W | Extracellular vesicle-derived LINC00482 induces microglial M2 polarization to facilitate brain metastasis of NSCLC | Cancer Lett | 2023 |
| 37040163 | Heo W | Triple-Negative Breast Cancer-Derived Extracellular Vesicles Promote a Hepatic Premetastatic Niche via a Cascade of Microenvironment Remodeling | Mol Cancer Res | 2023 |
| 37056561 | Wang Y | Tumor-derived Cav-1 promotes pre-metastatic niche formation and lung metastasis in breast cancer | Theranostics | 2023 |
| 37088585 | Kimoto A | Exosomes in ascites from patients with human pancreatic cancer enhance remote metastasis partially through endothelial-mesenchymal transition | Pancreatology | 2023 |
| 37194998 | Blavier L | The capture of extracellular vesicles endogenously released by xenotransplanted tumours induces an inflammatory reaction in the premetastatic niche | J Extracell Vesicles | 2023 |
| 37268941 | Zhang Z | Local radiotherapy for murine breast cancer increases risk of metastasis by promoting the recruitment of M-MDSCs in lung | Cancer Cell Int | 2023 |
| 37482043 | Han N | Cancer cell-derived extracellular vesicles drive pre-metastatic niche formation of lymph node via IFNGR1/JAK1/STAT1-activated-PD-L1 expression on FRCs in head and neck cancer | Oral Oncol | 2023 |
| 37563798 | Su X | Tumour extracellular vesicles induce neutrophil extracellular traps to promote lymph node metastasis | J Extracell Vesicles | 2023 |
| 37716915 | Horie M | Exosomes secreted by ST3GAL5(high) cancer cells promote peritoneal dissemination by establishing a premetastatic microenvironment | Mol Oncol | 2024 |
| 37759347 | Rigg E | Inhibition of extracellular vesicle-derived miR-146a-5p decreases progression of melanoma brain metastasis via Notch pathway dysregulation in astrocytes | J Extracell Vesicles | 2023 |
| 37781522 | Jia W | Hypoxia-induced exosomes facilitate lung pre-metastatic niche formation in hepatocellular carcinoma through the miR-4508-RFX1-IL17A-p38 MAPK-NF-κB pathway | Int J Biol Sci | 2023 |
| 37922311 | Dudgeon C | Netrin-1 feedforward mechanism promotes pancreatic cancer liver metastasis via hepatic stellate cell activation, retinoid, and ELF3 signaling | Cell Rep | 2023 |
| 37971863 | Mary B | Blood flow diverts extracellular vesicles from endothelial degradative compartments to promote angiogenesis | EMBO Rep | 2023 |
| 38215051 | Wu K | Tumor-Derived RAB21+ABHD12+ sEVs Drive the Premetastatic Microenvironment in the Lung | Cancer Immunol Res | 2024 |
| 38233863 | Gong L | Tumor-derived small extracellular vesicles facilitate omental metastasis of ovarian cancer by triggering activation of mesenchymal stem cells | Cell Commun Signal | 2024 |
| 38264628 | Sanchez VC | Crosstalk between tumor and stroma modifies CLIC4 cargo in extracellular vesicles | J Extracell Biol | 2023 |
| 38341855 | Deng C | Extracellular-vesicle-packaged S100A11 from osteosarcoma cells mediates lung premetastatic niche formation by recruiting gMDSCs | Cell Rep | 2024 |
| 38387275 | Jia W | Oleanolic acid inhibits hypoxic tumor-derived exosomes-induced premetastatic niche formation in hepatocellular carcinoma by targeting ERK1/2-NFκB signaling | Phytomedicine | 2024 |
| 38405101 | Ortiz A | NUPR1 packaged in extracellular vesicles promotes murine triple-negative breast cancer in a type 1 interferon-independent manner | Extracell Vesicles Circ Nucl Acids | 2024 |
| 38413999 | Li K | Tumor-derived exosomal ADAM17 promotes pre-metastatic niche formation by enhancing vascular permeability in colorectal cancer | J Exp Clin Cancer Res | 2024 |
| 38455075 | Brown TJ | Modulation of the pre-metastatic bone niche: molecular changes mediated by bone-homing prostate cancer extracellular vesicles | Front Cell Dev Biol | 2024 |
| 38472174 | Mao Y | Hypoxia induced exosomal Circ-ZNF609 promotes pre-metastatic niche formation and cancer progression via miR-150-5p/VEGFA and HuR/ZO-1 axes in esophageal squamous cell carcinoma | Cell Death Discov | 2024 |
| 38639925 | Jin J | NSCLC Extracellular Vesicles Containing miR-374a-5p Promote Leptomeningeal Metastasis by Influencing Blood‒Brain Barrier Permeability | Mol Cancer Res | 2024 |
| 38840551 | Mu W | Exposure of benzo[a]pyrene induces HCC exosome-circular RNA to activate lung fibroblasts and trigger organotropic metastasis | Cancer Commun (Lond) | 2024 |
| 38904015 | Lu F | Hypoxic tumor-derived exosomal miR-4488 induces macrophage M2 polarization to promote liver metastasis of pancreatic neuroendocrine neoplasm through RTN3/FABP5 mediated fatty acid oxidation | Int J Biol Sci | 2024 |
| 38907233 | Lin S | Cargo-eliminated osteosarcoma-derived small extracellular vesicles mediating competitive cellular uptake for inhibiting pulmonary metastasis of osteosarcoma | J Nanobiotechnology | 2024 |
| 38969706 | Roberts BK | IRF5 suppresses metastasis through the regulation of tumor-derived extracellular vesicles and pre-metastatic niche formation | Sci Rep | 2024 |
| 39136603 | Qiu R | ITGB3-enriched extracellular vesicles mediate the formation of osteoclastic pre-metastatic niche to promote lung adenocarcinoma bone metastasis | Mol Carcinog | 2024 |
| 39164758 | Zhou J | Targeting circ-0034880-enriched tumor extracellular vesicles to impede SPP1(high)CD206(+) pro-tumor macrophages mediated pre-metastatic niche formation in colorectal cancer liver metastasis | Mol Cancer | 2024 |
| 39329462 | Lin Y | Integrin α6-containing extracellular vesicles promote lymphatic remodelling for pre-metastatic niche formation in lymph nodes via interplay with CD151 | J Extracell Vesicles | 2024 |
| 39494337 | Lin Q | Breast cancer-derived CAV1 promotes lung metastasis by regulating integrin α6β4 and the recruitment and polarization of tumor-associated neutrophils | Int J Biol Sci | 2024 |
| 39497621 | Wei X | A switch from lysosomal degradation to secretory autophagy initiates osteogenic bone metastasis in prostate cancer | J Extracell Vesicles | 2024 |
| 39528717 | Yao Y | LSD1 deficiency in breast cancer cells promotes the formation of pre-metastatic niches | NPJ Precis Oncol | 2024 |
| 39630109 | Zhang L | Chronic Stress-Induced and Tumor Derived SP1(+) Exosomes Polarizing IL-1β(+) Neutrophils to Increase Lung Metastasis of Breast Cancer | Adv Sci (Weinh) | 2025 |
| 39633064 | Tang T | Breast cancer stem cell-derived exosomal lnc-PDGFD induces fibroblast-niche formation and promotes lung metastasis | Oncogene | 2025 |
| 39658708 | Chen Y | Tumor exosomal RNPEP promotes lung metastasis of liver cancer via inducing cancer-associated fibroblast activation | Cancer Sci | 2025 |
| 39710056 | Dong Q | Salivary adenoid cystic carcinoma-derived α2,6-sialylated extracellular vesicles increase vascular permeability by triggering ER-stress in endothelial cells and promote lung metastasis | Cancer Lett | 2024 |
| 39715212 | Fietta A | Neuroblastoma-derived hypoxic extracellular vesicles promote metastatic dissemination in a zebrafish model | PLoS One | 2024 |
| 39847320 | Zhang Y | Complement C3 of tumor-derived extracellular vesicles promotes metastasis of RCC via recruitment of immunosuppressive myeloid cells | Proc Natl Acad Sci U S A | 2025 |
| 39868462 | Busatto S | Breast Cancer-Derived Extracellular Vesicles Modulate the Cytoplasmic and Cytoskeletal Dynamics of Blood-Brain Barrier Endothelial Cells | J Extracell Vesicles | 2025 |
| 40055530 | Huang G | Role of GPX3+ astrocytes in breast cancer brain metastasis activated by circulating tumor cell exosomes | NPJ Precis Oncol | 2025 |
| 40120799 | Jiang C | Lactate accumulation drives hepatocellular carcinoma metastasis through facilitating tumor-derived exosome biogenesis by Rab7A lactylation | Cancer Lett | 2025 |
| 40134019 | Li T | Colorectal cancer cells-derived exosomal miR-188-3p promotes liver metastasis by creating a pre-metastatic niche via activation of hepatic stellate cells | J Transl Med | 2025 |
| 40234961 | Li S | Colon cancer exosome-associated HSP90B1 initiates pre-metastatic niche formation in the liver by polarizing M1 macrophage into M2 phenotype | Biol Direct | 2025 |

**Table 1. List of peer reviewed publications reporting a contribution of extracellular vesicles to metastasis**

This table lists publications from 2010 to 2025 exploring the role of extracellular vesicles in metastasis. Studies were restricted solely to work performed *in vivo*. Detailed search engine and data extraction process used to compile this table can be found in “Material and Methods” section. Columns indicate PMID, first author, title, journal/book and year of publication.
