# Supplementary material for: Contribution of tumor-derived extracellular vesicles in the establishment of the pre-metastatic niche: lessons learned from past experimentations and future directions
Source: Clin Exp Metastasis. 2026 Mar 7;43(2):16. doi: 10.1007/s10585-026-10396-z (PMC12967406; doi:10.1007/s10585-026-10396-z)
Supplement: Supplementary file 2 — Supplementary Material 2 [file 10585_2026_10396_MOESM2_ESM.docx]

| **Cancer Type** | **Lung** | **Liver** | **Bone** | **Lymphnode** | **Brain** | **peritoneal** | **Bone marrow** | **Kidney** | **Stomach** |
| --- | --- | --- | --- | --- | --- | --- | --- | --- | --- |
| **Exogenous models** |  |  |  |  |  |  |  |  |  |
| **Breast** | X | X | X |  | X |  |  |  |  |
| **Colon** | X | X |  | X |  |  |  |  |  |
| **Melanoma** | X |  |  | X | X |  |  |  | X |
| **Lung** | X | X |  |  | X |  | X |  |  |
| **Prostate** | X |  | X | X |  |  | X | X |  |
| **Liver** | X |  |  |  |  |  |  |  |  |
| **Pancreas** | X | X |  |  |  |  |  |  |  |
| **Osteosarcoma** | X |  |  |  |  |  |  |  |  |
| **Gastric** | X | X |  | X |  | X |  |  |  |
| **Salivary** | X |  |  |  |  |  |  |  |  |
| **Head and Neck** | X |  |  | X |  |  |  |  |  |
| **Nasopharyngeal** | X |  |  |  |  |  |  |  |  |
| **Ovary** |  |  |  |  |  | X |  |  |  |
| **Neuroblastoma** |  | X |  |  |  |  |  |  |  |
| **Esophagus** | X | X |  |  | X |  |  |  |  |
| **Bladder** | X | X |  | X |  |  |  |  |  |
| **Kidney** | X | X |  |  |  |  |  |  |  |
|  |  |  |  |  |  |  |  |  |  |
| **Endogenous models** |  |  |  |  |  |  |  |  |  |
| **Breast** | X |  |  |  |  |  |  |  |  |
| **Melanoma** | X |  |  |  |  |  |  |  |  |
| **Neuroblastoma** |  | X |  |  |  |  |  |  |  |
| **Prostate** |  |  | X |  |  |  |  |  |  |

**Supplemental Table 2.** Cancer types being studied in the papers selected through our search query and the metastatic sites being examined in exogenous and endogenous models.
