# Supplementary material for: Contribution of tumor-derived extracellular vesicles in the establishment of the pre-metastatic niche: lessons learned from past experimentations and future directions
Source: Clin Exp Metastasis. 2026 Mar 7;43(2):16. doi: 10.1007/s10585-026-10396-z (PMC12967406; doi:10.1007/s10585-026-10396-z)
Supplement: Supplementary file 3 — Supplementary Material 3 [file 10585_2026_10396_MOESM3_ESM.docx]

| **Fig.1b** |  |  |  |
| --- | --- | --- | --- |
| **Cancer** | % | **Metastasis** | % |
| Breast | 21.5 | Lung | 45.7 |
| Pancreas | 15.4 | Liver | 20.0 |
| Colon | 13.1 | Bone | 9.5 |
| Melanoma | 11.5 | Lymph node | 9.5 |
| Lung | 7.7 | Brain | 9.5 |
| Prostate | 6.2 | Bone Marrow | 2.9 |
| Liver | 6.2 | Peritoneal | 1.9 |
| Osteosarcoma | 4.6 | Kidney | 1.0 |
| Gastric | 4.6 | Omental | 1.0 |
| Salivary | 2.3 |  |  |
| Head and Neck | 2.3 |  |  |
| Ovary | 2.3 |  |  |
| Neuroblastoma | 1.5 |  |  |
| Bladder | 0.8 |  |  |
| Kidney | 0.8 |  |  |

| **Fig.1c** |  |  |  |  |  |  |  |
| --- | --- | --- | --- | --- | --- | --- | --- |
| **EV Purification** | % | **EV Characterization** | % | **EV**  **Labeling** | % | **EV Administration** | % |
| DUC | 56.0 | TEM | 31.0 | PKH28/67 | 36.3 | i.v. | 67.6 |
| UC | 22.0 | NTA | 27.0 | DiD/DiI/DiO/DiR | 24.8 | r.o. | 9.5 |
| DGC | 6.0 | WB | 9.1 | Cy3/5/5.5/7 | 5.3 | i.f.p. | 6.7 |
| DUC+DGC | 7.8 | TEM/NTA/WB | 24.0 | CD63-GFP/RFP/pHluorin/mRuby | 5.3 | i.p. | 5.7 |
| Exoquick | 9.2 | Flow | 2.8 | miR | 3.5 | i.c. | 1.9 |
| SEC | 4.3 | Proteomics | 2.8 | CSFE | 1.8 | i.cr | 1.9 |
| DUC+SEC | 0.9 | Unmentioned | 2.8 | Others | 4.4 | s.c. | 0.95 |
| Ultrafiltration | 2.5 |  |  | Unlabeled | 14.2 | i.e. | 0.95 |
|  |  |  |  |  |  | i.b.m. | 0.95 |
|  |  |  |  |  |  | i.a | 0.95 |
|  |  |  |  |  |  | i.plug | 0.95 |
|  |  |  |  |  |  | i.tumor | 0.95 |
|  |  |  |  |  |  | perfusion | 0.95 |

| **Fig.1f** |  |
| --- | --- |
| **Mice** | % |
| Immunocompromised | 55.4 |
| Immunocompetent | 44.6 |
|  |  |
| Nude | 41.8 |
| SCID | 7.0 |
| NOD | 6.2 |
| C57Bl/6 | 24.8 |
| Balb/c | 18.6 |
| FVB | 1.6 |

| **Fig.1g** |  |  |  |
| --- | --- | --- | --- |
| **TEV-capturing cells** | % |  | % |
| Immunocompromised |  | Immunocompetent |  |
| Fibroblasts | 21.3 |  | 21.5 |
| Endothelial Cells | 14.7 |  | 12.3 |
| Lymphatic Endothelial | 4.0 |  | 6.2 |
| Macrophages | 14.7 |  | 27.7 |
| Kupffer Cells | 5.5 |  | 3.1 |
| Hepatic Stellate Cells | N/A |  | 3.1 |
| Epithelial Cells | 5.3 |  | 1.5 |
| Osteoclasts | 5.3 |  | N/A |
| Osteoblasts | 4.0 |  | 1.5 |
| Myeloid | 2.7 |  | 3.1 |
| Mesenchymal Stem Cells | 2.7 |  | 1.5 |
| Natural Killer | N/A |  | 1.5 |
| Neutrophils | N/A |  | 3.1 |
| Astrocytes | 2.7 |  | 3.1 |
| Tumor Cells | 1.3 |  | N/A |
| Others | 1.3 |  | 1.5 |
| ND | 16.0 |  | 9.2 |

| **Fig.1h** |  |
| --- | --- |
| **Effect of TEV Uptake** | % |
| Immune Reprogramming | 30.8 |
| Stromal Cell Activation | 24.3 |
| Angiogenesis/Vascular Permeability | 19.6 |
| Lymphangiogenesis | 7.5 |
| Bone Remodeling | 9.3 |
| ECM Remodeling | 8.4 |
| Tumorigenicity | 2.8 |
